# GBLUP Outperforms Quantile Mapping and Outlier Detection for Enhanced Genomic Prediction

**DOI:** 10.3390/ijms26083620

**Published:** 2025-04-11

**Authors:** Osval Antonio Montesinos-López, José Crossa, Paolo Vitale, Guillermo Gerard, Leonardo Crespo-Herrera, Susanne Dreisigacker, Carolina Saint Pierre, Luis G. Posadas, Afolabi Agbona, Raymundo Buenrostro-Mariscal, Abelardo Montesinos-López, Aakash Chawade

**Affiliations:** 1 Facultad de Telemática, Universidad de Colima, Colima 28040, CL, Mexico; osval78t@gmail.org (O.A.M.-L.); r.buenrostro@ucol.mx (R.B.-M.); 2 International Maize and Wheat Improvement Center (CIMMYT), Km 45, Carretera México–Veracruz, Texcoco 52640, EM, Mexico; j.crossa@cgiar.org (J.C.); p.vitale@cgiar.org (P.V.); g.gerard@cgiar.org (G.G.); l.crespo@cgiar.org (L.C.-H.); s.dreisigacker@cgiar.org (S.D.); c.saintpierre@cgiar.org (C.S.P.); 3 Colegio de Postgraduados, Montecillos 56230, EM, Mexico; 4 Department of Agronomy and Horticulture, University of Nebraska, 363 Keim Hall, Lincoln, NE 68583, USA; l.posadas@ucol.com.ma; 5 International Institute of Tropical Agriculture (IITA), Ibadan 200001, Nigeria; 6 Molecular & Environmental Plant Sciences, Texas A&M University, College Station, TX 77843, USA; 7 Centro Universitario de Ciencias Exactas e Ingenierías (CUCEI), Universidad de Guadalajara, Guadalajara 44430, JA, Mexico; 8 Department of Plant Breeding at SLU, Swedish University of Agricultural Sciences, P.O. Box 190 Alnarp, SE-23422 Lomma, Sweden

**Keywords:** quantile mapping, GBLUP, outlier detection methods, plant breeding, genomic prediction

## Abstract

Genomic selection (GS) accelerates plant breeding by predicting complex traits using genomic data. This study compares genomic best linear unbiased prediction (GBLUP), quantile mapping (QM)—an adjustment to GBLUP predictions—and four outlier detection methods. Using 14 real datasets, predictive accuracy was evaluated with Pearson’s correlation (COR) and normalized root mean square error (NRMSE). GBLUP consistently outperformed all other methods, achieving an average COR of 0.65 and an NRMSE reduction of up to 10% compared to alternative approaches. The proportion of detected outliers was low (<7%), and their removal had minimal impact on GBLUP’s predictive performance. QM provided slight improvements in datasets with skewed distributions but showed no significant advantage in well-distributed data. These findings confirm GBLUP’s robustness and reliability, suggesting limited utility for QM when data deviations are minimal.

## 1. Introduction

Genomic selection (GS) has changed plant breeding over the past decade, fundamentally transforming genetic evaluation and selection. By integrating genomic data into predictive models, GS has accelerated breeding cycles, improved selection precision, and enhanced genetic gains [1,2]. Unlike traditional methods reliant on extensive phenotypic evaluations, GS leverages genome-wide markers to predict genotype performance, reducing the costs and time associated with field trials [3]. This innovation has been pivotal in addressing global challenges such as food security and climate change by enabling the rapid development of high-yielding, resilient crop varieties [4]. Today, GS is a cornerstone of modern plant breeding, integrating cutting-edge technologies and big data analytics to drive sustainability and innovation.

GS has been successfully applied across diverse crops, enhancing yield potential and disease resistance in maize and wheat [2], accelerating the development of stress-tolerant rice varieties [5], and shortening breeding cycles in perennials like sugarcane and oil palm [6]. Its ability to predict genetic potential using genome-wide markers has significantly reduced the need for extensive phenotypic evaluations. Additionally, GS has improved genetic gains for complex traits such as drought tolerance and nutrient use efficiency, underscoring its transformative impact on modern agriculture [7].

The GBLUP (genomic best linear unbiased prediction) statistical model remains one of the most popular and widely used approaches in genomic prediction due to its simplicity, robustness, and interpretability. Despite the emergence of modern machine learning methods, GBLUP is preferred in many cases because it is computationally efficient and provides reliable predictions, especially for traits controlled by many small-effect loci [8]. Its linear mixed-model framework accounts for genetic relationships using genomic relationship matrices, making it particularly suitable for plant and animal breeding programs [1]. While machine learning methods like random forests and deep learning can capture complex non-linear interactions, they often require large datasets, extensive hyperparameter tuning, and are prone to overfitting when data are limited [2]. In contrast, GBLUP provides a balance between accuracy and simplicity, ensuring stable performance across a variety of traits and environments [9,10]. Its widespread adoption by GS underscores its reliability and practical advantages, particularly in agricultural contexts where interpretability and computational feasibility are critical.

Given the computational efficiency and widespread use of GBLUP in genomic prediction, there is significant interest in exploring strategies to enhance its predictive power. Combining GBLUP with quantile mapping (QM) and outlier detection techniques offers a promising avenue for improvement. Quantile mapping can address biases in the distribution of predicted values by aligning them more closely with the observed data, thereby increasing prediction accuracy and ensuring a better calibration [11]. Outlier detection, on the other hand, enhances the robustness of the model by identifying and removing data points that disproportionately influence predictions, which is especially crucial in genomic datasets prone to noise and inconsistencies [12]. Together, these methods can, in theory, synergistically improve GBLUP by refining its inputs and outputs, ultimately leading to more reliable predictions. This combined approach not only leverages the interpretability and computational advantages of GBLUP but also integrates advanced techniques to address limitations inherent to genomic datasets, making it a powerful tool for plant and animal breeding.

QM is widely utilized across disciplines for bias correction and improving data alignment. In climate science, QM adjusts biases in model outputs, enhancing the accuracy of temperature and precipitation projections for reliable climate assessments [13]. In hydrology, it refines streamflow and rainfall-runoff predictions, crucial for flood and drought evaluations [14]. In remote sensing, QM harmonizes satellite-derived data with ground-based observations, improving environmental dataset utility [15]. Beyond environmental sciences, QM is applied in genomics for aligning predicted values with observed data, enhancing prediction accuracy, and in economics for bias correction in income and risk assessments. Its versatility makes QM a critical tool across multiple fields.

Outlier detection plays a critical role in improving predictions in machine learning by identifying and mitigating the impact of anomalous data points that can distort model performance. By detecting and removing outliers, models achieve a better generalization, reduced bias, and enhanced accuracy, especially in regression and classification tasks. Methods such as statistical thresholds, clustering, and advanced algorithms like isolation forests are commonly applied to detect outliers in diverse datasets. Outlier detection has shown effectiveness in applications such as genomic prediction, fraud detection, and environmental modeling, where precise predictions are essential for decision-making [16]. These approaches refine training data quality and ultimately lead to more robust and reliable machine learning models [17,18]. These studies underscore the importance of addressing outliers to enhance the reliability of genomic prediction models.

As already mentioned, previous studies have shown that quantile mapping (QM) and outlier detection can enhance GBLUP for genomic predictions, which motivated this study. QM improves calibration by aligning predicted values with observed distributions, addressing biases from GBLUP’s normality assumptions. Outlier detection enhances robustness by mitigating the impact of extreme values that could distort variance estimates and bias predictions. Given these prior findings, this study aimed to further evaluate their effectiveness. To strengthen the rationale, it is important to explicitly reference previous studies, clarify how these methods theoretically improve predictions, and demonstrate their impact through comparative analyses.

By leveraging QM for bias correction and four outlier detection methods (Invchi, Logit, Meanp, and SumZ) to refine the training set, this study aims to maximize the predictive potential of GBLUP across diverse datasets. The benchmark analysis, conducted on 14 real datasets, evaluates predictive accuracy using Pearson’s correlation (COR) and normalized mean square error (NRMSE), showcasing the synergistic effects of combining these complementary methods. However, for simplicity, we present full results below for three datasets, Disease, EYT_1, and Wheat_1, as well as results across datasets. We studied GBLUP alone and GBLUP in combination with quantile mapping (QM) and four outlier detection models (Invchi, Logit, Meanp, and SumZ) making a total of 10 genomic prediction models. Several results for datasets are shown in Appendix A, Appendix B and Appendix C.

## 2. Results

The results are presented in four sections. Section 1, Section 2 and Section 3 present the results for the datasets Disease, EYT_1, and Wheat_1. Section 4 provides the results across datasets. Appendix A provides the tables of results corresponding to datasets Disease, EYT_1, Wheat_1, and across datasets. Appendix B and Appendix Cprovide the figures and tables of results for the other datasets included in the study: Maize, Japonica, Indica, Groundnut, EYT_2, EYT_3, Wheat_2, Wheat_3, Wheat_4, Wheat_5, and Wheat_6. The results are provided in terms of the metrics of Pearson’s correlation (COR) and normalized mean square error (NRMSE). The assignment of datasets to the appendices was random, that is, not based on any specific criteria.

As described in the Section 4 below, we compared the genomic prediction accuracy of 10 different model options: GBLUP alone; GBLUP combined only with quantile mapping (QM); GBLUP combined with the four outlier detection methods (Invchi, Logit, Meanp, and SumZ); and GBLUP combined with the four combinations of quantile mapping (QM) with the four outlier detection methods (QM_Invchi, QM_Logit, QM_Meanp, and QM_SumZ).

### 2.1. Disease

**Figure 1:**
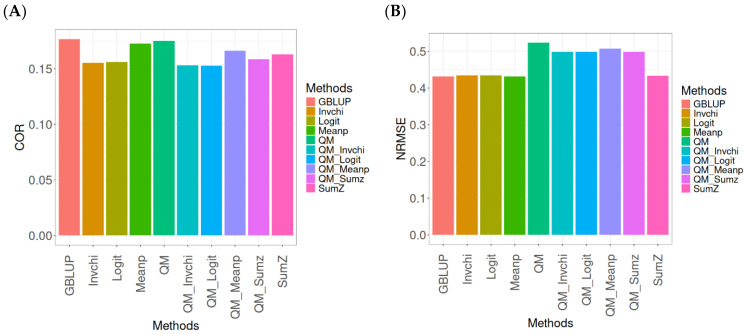
Comparative performance of genomic prediction methods in terms of Pearson’s correlation (COR) (**A**) and normalized root mean square error (NRMSE) (**B**) for Disease dataset.

Figure 1 presents the results for the Disease dataset under a comparative analysis of the GBLUP, Invchi, Logit, Meanp, Sumz, QM, QM_Invchi, QM_Logit, QM_Meanp, QM_Sumz, and Sumz models in terms of their predictive efficiency measured by COR and NRMSE. For more details, see Table A1 (in Appendix A).

The analysis of Pearson’s correlation between observed and predicted values (Figure 1A) for the Disease dataset reveals that the GBLUP method stands out as the most effective approach, achieving a correlation of 0.1766, which is 0.8567% greater than QM’s correlation of 0.1751. In comparison to other methods, GBLUP significantly outperforms Meanp (0.1728, 2.1991% less effective), QM_Meanp (0.1661, 6.3215% less effective), SumZ (0.1630, 8.3436% less effective), QM_Sumz (0.1586, 11.3493% less effective), Logit (0.1559, 13.2777% less effective), Invchi (0.1552, 13.7887% less effective), QM_Invchi (0.1530, 15.4248% less effective), and QM_Logit (0.1528, 15.5759% less effective).

Regarding the NRMSE metric between observed and predicted values (Figure 1B) for the Disease dataset, the results indicate that the GBLUP method achieves the lowest average NRMSE, making it the most effective option. GBLUP yields a value of 0.4313, which is 0.1159% better than Meanp (0.4318) and 0.5565% better than SumZ (0.4337). Additionally, GBLUP outperforms Logit (0.4345) by 0.7419% and Invchi (0.4346) by 0.7651%. Notably, GBLUP also shows significant advantages over QM_Logit (0.4984) by 15.5576%, QM_Invchi (0.4986) by 15.604%, QM_Sumz (0.4987) by 15.6272%, QM_Meanp (0.5072) by 17.598%, and QM (0.5234) by 21.354%.

Overall, the analysis of the Disease dataset indicates that the GBLUP method is the most effective approach, demonstrating a higher Pearson’s correlation compared to other methods, including QM and Meanp. This trend is also reflected in the NRMSE metric, where GBLUP achieves the lowest average NRMSE, confirming its superior performance. Its advantages over a range of alternative methods, including various quantile mapping strategies, further solidify the reliability and effectiveness of GBLUP for predictive tasks in this context.

### 2.2. EYT_1

**Figure 2:**
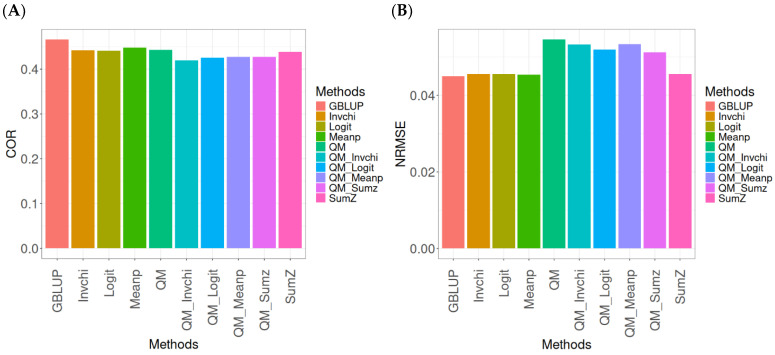
Comparative performance of genomic prediction methods in terms of Pearson’s correlation (COR) (**A**) and normalized root mean square error (NRMSE) (**B**) for EYT_1 dataset.

The results for the models evaluated on the EYT_1 dataset (Figure 2) were assessed using the same metrics, COR and NRMSE. For more details, see Table A2 (in Appendix A).

The evaluation of Pearson’s correlation between observed and predicted values (Figure 2A) for the EYT_1 dataset indicates that the GBLUP method emerges as the most effective strategy, attaining a correlation of 0.4659, which is 3.9955% greater than Meanp’s correlation of 0.4480. In relation to other approaches, GBLUP significantly surpasses QM (0.4429, 5.193% less effective), Invchi (0.4417, 5.4788% less effective), Logit (0.4414, 5.5505% less effective), SumZ (0.4389, 6.1517% less effective), QM_Meanp (0.4273, 9.0335% less effective), QM_Sumz (0.4270, 9.1101% less effective), QM_Logit (0.4257, 9.4433% less effective), and QM_Invchi (0.4193, 11.1138% less effective).

Regarding the NRMSE metric between observed and predicted values (Figure 2B) for the EYT_1 dataset, the findings reveal that the GBLUP method achieves the lowest average NRMSE, establishing it as the most effective choice. GBLUP has a value of 0.0450, which is 0.8889% greater than Meanp (0.0454) and 1.1111% better than Invchi (0.0455). Additionally, GBLUP outperforms Logit (0.0456) and SumZ (0.0456) by 1.3333%. Notably, GBLUP also exhibits significant advantages over QM_Sumz (0.0512) by 13.7778%, QM_Logit (0.0519) by 15.3333%, QM_Invchi (0.0533) by 18.4444%, QM_Meanp (0.0534) by 18.6667%, and QM (0.0545) by 21.1111%.

Overall, the analysis of the EYT_1 dataset indicates that the GBLUP method consistently outperforms other strategies, displaying both the highest Pearson’s correlation and the lowest NRMSE. This establishes GBLUP as the most effective choice compared to Meanp, Invchi, and the various quantile mapping methods. Its superior performance across both metrics underscores its reliability and potential for the enhancement of predictive accuracy in related applications.

### 2.3. Wheat_1

This section presents the results of the genomic prediction models evaluated on the Wheat_1 data, considering the same metrics as before. For more details, see Table A3 (in Appendix A).

**Figure 3:**
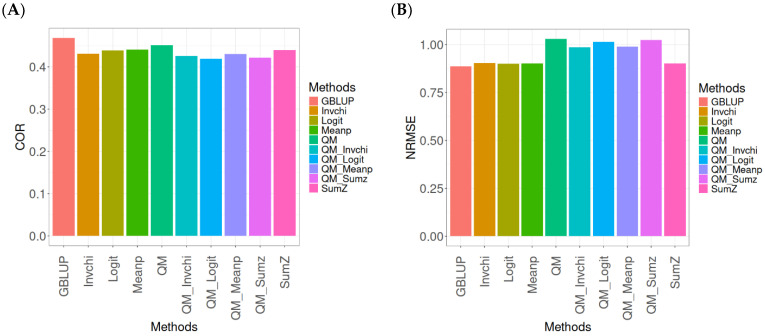
Comparative performance of genomic prediction methods in terms of Pearson’s correlation (COR) (**A**) and normalized root mean square error (NRMSE) (**B**) for Wheat_1 dataset.

The assessment of Pearson’s correlation between observed and predicted values (Figure 3A) for the Wheat_1 dataset shows that the GBLUP method emerges as the most effective strategy, achieving a correlation of 0.4682, which is 3.8598% greater than Meanp’s correlation of 0.4406. In comparison to other methods, GBLUP significantly outperforms QM (0.4508, 6.2642% less effective), SumZ (0.4400, 6.4091% less effective), Logit (0.4387, 6.7244% less effective), Invchi (0.4314, 8.5304% less effective), QM_Meanp (0.4299, 8.909% less effective), QM_Invchi (0.4256, 10.0094% less effective), QM_Sumz (0.4214, 11.1058% less effective), and QM_Logit (0.4187, 11.8223% less effective).

Regarding the NRMSE metric between observed and predicted values (Figure 3B) for the Wheat_1 dataset, the findings indicate that the GBLUP method achieves the lowest average NRMSE, establishing it as the most effective option. GBLUP has a value of 0.887, which is 1.5671% better than Logit (0.9009) and 1.6347% greater than Meanp (0.9015). Additionally, GBLUP outperforms SumZ (0.9016) by 1.646% and Invchi (0.9047) by 1.9955%. Notably, GBLUP also presents significant advantages over QM_Invchi (0.9866) by 11.2289%, QM_Meanp (0.9895) by 11.5558%, QM_Logit (1.0148) by 14.4081%, QM_Sumz (1.0238) by 15.4228%, and QM (1.0293) by 16.0428%.

The assessment of the Wheat_1 dataset reveals that the GBLUP method is the most effective strategy, achieving a higher Pearson’s correlation compared to other approaches, including Meanp and remaining methods. The performance of GBLUP is not only superior in correlation but also presents the lowest average NRMSE, further establishing its effectiveness. It significantly outperforms other methods, such as Logit and SumZ, as well as a range of quantile mapping strategies, indicating its reliability for predictive tasks. Overall, the consistent advantages of GBLUP reinforce its position as the preferred method in this context.

### 2.4. Across Data

In this section, the analysis of the results presented across datasets is given under the same model and metrics as before. For more details, see Table A4 (in Appendix A).

**Figure 4:**
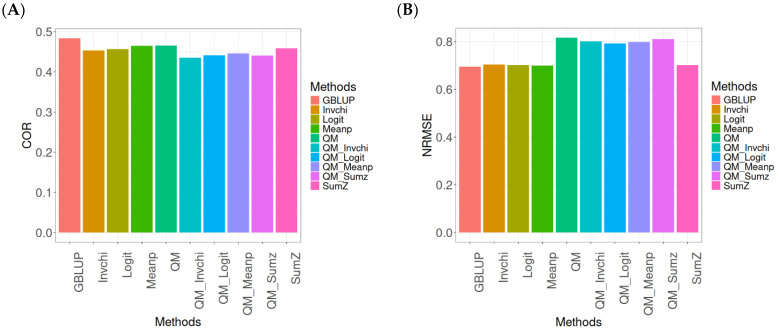
Comparative performance of genomic prediction methods in terms of Pearson’s correlation (COR) (**A**) and normalized root mean square error (NRMSE) (**B**) across datasets, using quantile mapping.

The assessment of Pearson’s correlation between observed and predicted values (Figure 4A) across datasets highlights the GBLUP method as the most effective strategy, achieving a correlation of 0.4834, which is 3.9794% greater than Meanp’s correlation of 0.4649. In comparison to other methods, GBLUP significantly outperforms QM (0.4659, 3.7562% less effective), SumZ (0.4584, 5.4538% less effective), Logit (0.4569, 5.8% less effective), and Invchi (0.4533, 6.6402% less effective). Notably, GBLUP also shows advantages over various quantile mapping methods, including QM_Meanp (0.4458, 8.4343% less effective), QM_Logit (0.4412, 9.5648% less effective), QM_Sumz (0.4405, 9.7389% less effective), and QM_Invchi (0.4355, 10.9989% less effective).

The assessment of the NRMSE metric between observed and predicted values (Figure 4B) across datasets indicates that the GBLUP method achieves the lowest average NRMSE, establishing it as the most effective option. GBLUP has a value of 0.6954, which is 0.7046% better than Meanp (0.7003) and 0.9347% greater than SumZ (0.7019). Additionally, GBLUP outperforms Logit (0.7019) and Invchi (0.7043) by 0.9347% and 1.2798%, respectively. Notably, GBLUP also presents significant advantages over various quantile mapping methods, including QM_Logit (0.7928) by 14.0063%, QM_Meanp (0.7976) by 14.6966%, QM_Invchi (0.8018) by 15.3005%, QM_Sumz (0.8110) by 16.6235%, and QM (0.8160) by 17.3425%.

The assessment of Pearson’s correlation across datasets reveals that the GBLUP method is the most effective approach, achieving a higher correlation compared to other methods, including Meanp and various quantile mapping strategies. GBLUP not only excels in correlation but also records the lowest average NRMSE, solidifying its status as the most reliable option. Its performance surpasses that of Logit and SumZ, as well as several quantile mapping methods, indicating a clear advantage. Overall, GBLUP’s consistent effectiveness across both metrics reinforces its preference for predictive tasks in this context.

## 3. Discussion

The successful implementation of GS in plant breeding faces several challenges, including the need for high-quality genomic and phenotypic data, appropriate statistical models, and robust validation strategies. One key hurdle is the limited availability of large, diverse datasets required to capture the genetic architecture of complex traits and account for genotype-by-environment interactions, which are critical in breeding programs targeting multiple environments [2,19]. Additionally, computational demands increase significantly with the inclusion of high-dimensional genomic data, requiring advancements in algorithms and computational resources. Another challenge lies in translating GP predictions into actionable breeding decisions, demanding integration with traditional breeding practices and decision-support tools [20]. Addressing these issues involves interdisciplinary collaboration and significant investment in training, data curation, and infrastructure to fully leverage the potential of GP in enhancing genetic gains and breeding efficiency.

Improving the efficiency of GS in plant breeding relies on strategies that enhance prediction accuracy, optimize resource allocation, and integrate GS into breeding pipelines. One successful approach is the use of multi-environment trials (MET) to capture genotype-by-environment interactions, enabling better predictions across diverse target environments [2]. Sparse testing schemes, which involve phenotyping only a subset of genotypes in certain environments, are also effective in reducing costs while maintaining prediction accuracy when paired with robust statistical models [21,22]. Additionally, leveraging complementary data sources such as high-throughput phenotyping and environmental covariates can further enhance GS accuracy by providing insights into complex trait architectures [23]. Implementing these strategies requires investment in advanced data management systems and interdisciplinary collaboration to fully integrate GS into breeding programs and maximize genetic gains.

Despite its potential, the practical application of GS in plant breeding remains highly challenging due to complexities such as the need for high-quality genomic and phenotypic data, the variability in genotype-by-environment interactions, and the computational burden of analyzing large datasets. The effectiveness of GS often depends on the accuracy of prediction models, which can be hindered by limited training data, especially for less-studied traits or environments [24]. Furthermore, the integration of GS into breeding programs requires adapting existing workflows and overcoming economic and logistical barriers, such as the cost of genotyping and the need for skilled personnel [2]. To address these limitations, researchers are actively exploring novel approaches, including integrating environmental data, leveraging machine learning techniques, and developing strategies like sparse testing to improve the efficiency and scalability of GS [25]. These efforts aim to refine GS methodologies and make them more applicable to real-world breeding scenarios.

For this reason, this study explored the use of quantile mapping and the removal of outlier observations within a GBLUP framework to improve the predictive accuracy of the conventional GBLUP model. In theory, these combinations have the potential to enhance the prediction accuracy of GBLUP by addressing critical issues such as the influence of extreme values and non-normality in the data. Quantile mapping, by transforming the distribution of predictions to better align with observed values, can correct systematic biases that often undermine model performance. Simultaneously, outlier removal helps reduce noise and ensures that the model focuses on patterns representative of the majority of the data, which is particularly important when dealing with genomic data characterized by high dimensionality and complex interactions. These adjustments aim to refine the training dataset and statistical assumptions of the model, ultimately resulting in more robust and reliable predictions. Furthermore, integrating these strategies within the GBLUP framework offers an opportunity to adapt this widely used genomic prediction method to varying data qualities and environmental conditions, addressing persistent challenges in plant breeding programs.

However, our results combining the GBLUP method with quantile mapping and outlier detection techniques did not meet expectations. In terms of Pearson’s correlation, across all datasets and within each individual dataset, the GBLUP method proved to be the most effective, consistently achieving higher correlations than the alternative approaches. This superior performance of GBLUP is further supported by its ability to minimize errors, as evidenced by lower NRMSE values. Compared to other methods, including any outlier detection method, quantile mapping, and resulting combinations of quantile mapping with outlier detection techniques, GBLUP consistently delivers more accurate predictions, reaffirming its reliability and robustness in the context of breeding programs.

Our results emphasize the benefits and robustness of the GBLUP method, which remains one of the most popular approaches for genomic prediction. Its popularity stems from several key factors. Firstly, GBLUP is computationally efficient and relatively simple to implement, making it accessible for a wide range of breeding programs. Secondly, it leverages genomic relationships to predict breeding values, effectively capturing additive genetic effects, which are crucial for many quantitative traits. Additionally, GBLUP is grounded in a solid statistical framework, offering reliable and interpretable results. Its ability to handle high-dimensional genomic data without overfitting further contributes to its widespread use. Moreover, the compatibility of GBLUP with extensions, such as the incorporation of environmental covariates or non-additive effects, enhances its adaptability to complex breeding scenarios. These advantages collectively solidify the position of GBLUP as a cornerstone method in genomic prediction.

Finally, we want to emphasize that our results are specific to the datasets used in this study, which reflect genetic and environmental conditions. The observed lack of improvement in predictive accuracy when combining GBLUP with quantile mapping and outlier detection techniques may be influenced by the nature of the datasets, such as their size, genetic architecture, or level of noise. While these combinations did not outperform the conventional GBLUP method in this context, it is important to acknowledge that their effectiveness could vary under different circumstances. For instance, in datasets with pronounced outliers or non-normal distributions, quantile mapping and outlier removal may play a more significant role in improving model performance. Additionally, these techniques might offer advantages in scenarios in which specific traits exhibit strong non-linear patterns or in which genotype-by-environment interactions are highly complex. Therefore, while our findings reaffirm the robustness of the standard GBLUP method, they also suggest the need for further exploration of these combinations across diverse datasets to fully understand their potential.

This study evaluates the impact of quantile mapping and outlier detection on the accuracy of genomic predictions using GBLUP. However, confidence intervals for accuracy metrics, such as Pearson’s correlation and root means square error, were not computed, which limits the ability to assess the statistical uncertainty associated with the observed improvements. Additionally, formal hypothesis testing, such as paired statistical tests to compare GBLUP with and without these enhancements, was not conducted. While the study primarily focused on practical predictive improvements rather than statistical inference, future research should incorporate bootstrapping or cross-validation techniques to estimate confidence intervals and apply appropriate statistical tests, such as paired *t*-tests or Wilcoxon signed-rank tests, to determine whether the observed differences are statistically significant. Implementing these approaches would strengthen the robustness of the conclusions and provide a clearer understanding of the reliability and generalizability of the proposed methods across different datasets and breeding populations.

Furthermore, computational time was not systematically evaluated, which is an important factor when implementing these methods in large-scale genomic selection programs. Future studies should assess the trade-off between improved prediction accuracy and the additional computational cost associated with quantile mapping and outlier detection, particularly in large datasets where efficiency is a key consideration. Implementing these approaches would strengthen the robustness of the conclusions and provide a clearer understanding of the reliability, scalability, and generalizability of the proposed methods across different datasets and breeding populations.

## 4. Methods and Materials

### 4.1. Datasets

A detailed overview of the 14 datasets used in this study is provided in Table 1.

### 4.2. Bayesian GBLUP Model

The GBLUP model implemented was:

(1) $$Y_{i}=\mu +g_{i}+\epsilon_{i}\;$$

where $Y_{i}$ represents the best linear unbiased estimates (BLUE) for the i-th genotype. The grand mean is denoted by μ, and the random effects associated with genotypes (Lines), $g_{j},$ j=1,…,J, is distributed as $g={\left(g_{1},\ldots ,g_{J}\right)}^{T}∼N_{J}\left(0,\sigma_{g}^{2}G\right)$, where G is the genomic relationship-matrix [8] and $\sigma_{g}^{2}$ is the genetic variance component. The residual errors, $\epsilon_{i},$ are assumed to be independent and normally distributed with mean 0 and variance $\sigma^{2}$. This model was implemented in R statistical software version 4.4.3 [26] with the BGLR library of Pérez and de los Campos [27].

### 4.3. Quantile Mapping (QM)

QM is a widely used bias adjustment technique for post-processing climate model simulations. It addresses the mismatch between the coarse spatial resolution of model outputs and finer spatial scales of interest [9]. QM achieves this by aligning the cumulative density function (CDF) of the modeled data with that of reference data for each target location. Specifically, it creates a quantile-dependent correction function to map simulated quantiles to their corresponding reference values. This correction function is then applied to the modeled time series, yielding bias-adjusted values that align with the distribution of the reference data. QM operates under the assumption that the CDF of a variable in the forecast and observational time series remains consistent in future periods [28]. Given variable *x*, QM minimizes discrepancies between the CDFs of model data and reference data over a calibration period. In practice, the algorithm maps the model output *x* to the observational output *y* using a transformation function ℎ, ensuring the two CDFs become equivalent [29]. In terms of equations, this results in:

$$y=h\left(x\right)\to CDF_{y}\left(y\right)=CDF_{x}(x)$$

(2) $$y={CDF_{y}}^{-1}(CDF_{x}\left(x\right))$$

where ${CDF}^{-1}$ is the inverse function of the CDF. From Equation (1) it becomes clear that QM equates the cumulative distribution functions (CDFs) $CDF_{y}$ and $CDF_{x},$ respectively, of the observed data y and modeled data x, over a historical period, which results in the transfer function (1). The implemented QM scheme was based on the R package map version 3.4.2.1 [13].

Since the QM method relies on the observed and predicted values from the training set to adjust predictions, it is important to emphasize that QM is specifically implemented to refine the predicted values generated by the GBLUP method. In other words, the conventional GBLUP results are enhanced through this QM adjustment process.

### 4.4. Outlier Detection Methods

The four methods used for the detection of influential measures are based on the *p*-value-based meta-analysis approach proposed by Budhlakoti and Mishra [30]. A brief description of these approaches is as follows. Let us assume there are K independent tests, and their corresponding *p*-values are $p_{1}$, $p_{2}$,…,$\;p_{k}$. Under $H_{0}$, it is assumed that *p*-values from different methods (for individual observations) are uniformly distributed between 0 and 1 (i.e., $p_{k}\sim U(0,1)$). To determine the overall statistical significance of the hypothesis under test ($H_{0},$ i.e., null hypothesis vs. $H_{1},$ alternative hypothesis), individual *p*-values for each observation/genotype from different methods are combined. The specific methods used for this purpose are summarized in Table 2.

This approach (Table 2) was used to compute the final statistical significance values, specifically the combined *p*-values for the selected observations or genotypes. Influential observations were determined by applying a suitable *p*-value threshold. The methods were implemented using source code available from a GitHub repository at GitHub—BudhlakotiN/OGS: R/OGS: Outlier in Genomics Data.

It is important to note that these four outlier detection methods (Invchi, Logit, Meanp, SumZ) were applied to the training set of each fold. After implementation, any observations identified as outliers were removed from the training set. The reduced training set was then used to implement the GBLUP method, as described in Equation (1).

### 4.5. Combining Quantile Mapping with Outlier Detection Methods Using GBLUP

Combining the quantile mapping (QM) method with the four outlier detection methods (Invchi, Logit, Meanp, and SumZ) resulted in the development of four additional approaches, denoted as QM_Invchi, QM_Logit, QM_Meanp, and QM_SumZ. These methods were implemented as follows: first, each of the four outlier detection methods was applied as previously described. Subsequently, the QM method was applied using the observed and predicted values from the training set produced by each of the four outlier detection methods.

Therefore, a total of 10 models were employed in this study. These included: GBLUP alone; GBLUP combined with quantile mapping (QM); GBLUP combined with the four outlier detection methods, Invchi, Logit, Meanp, and SumZ; and, finally, GBLUP combined with the four combinations of quantile mapping (QM) with the outlier detection methods (QM_Invchi, QM_Logit, QM_Meanp, and QM_SumZ). Results are thus presented for a total of 10 combinations of GBLUP-based models, incorporating various combinations with QM and outlier detection methods.

### 4.6. Evaluation of Prediction Performance

To evaluate the proposed methods, we used cross-validation; more specifically, a 10-fold cross-validation approach. In each fold, 80% of the data were allocated for training and 20% for testing. For each testing set, prediction accuracy was assessed using two metrics: average Pearson’s correlation (COR) and normalized root mean square error (NRMSE) [35]. These metrics were selected because they facilitate comparisons across different traits, being independent of the trait’s scale. Both metrics were calculated using the observed values ($y_{i}$) and the predicted values [$\hat{f}(x_{i})$] from the testing set of each fold. The average performance over the 10 folds was reported. COR and NRMSE were selected not only for their utility in genomic prediction but also because they are widely used metrics for the evaluation of prediction performance.

## 5. Conclusions

Our benchmark analysis shows that the conventional GBLUP method outperforms quantile mapping, outlier detection techniques, and their combination in the context of genomic prediction. These findings reaffirm the effectiveness and robustness of GBLUP, which remains one of the most widely used techniques in plant and animal breeding for genomic selection. However, our results are not definitive, as substantial empirical evidence suggests that removing outliers from the training data can enhance prediction accuracy and quantile mapping can improve predictions in the testing set. Therefore, further empirical evaluations are essential to thoroughly assess the benefits and limitations of these alternative methods within the context of genomic selection. This will provide a more comprehensive understanding of their potential to complement or improve upon GBLUP.

## Figures and Tables

**Table 1:** Brief data description. RCBD denotes randomized complete block design, while alpha-lattice denotes the alpha lattice experimental design.

Data	No. Lines	No. Markers	Multi-Environment Data	BLUEs Across Environments	Experimental Design
Indica	327	16,383	YES	YES	RCBD
Japonica	320	16,383	YES	YES	RCBD
Groundnut	318	8268	YES	YES	Alpha-lattice
Maize	722	54,113	YES	YES	RCBD
Wheat_1	1301	78,606	YES	YES	Alpha-lattice
Wheat_2	1403	78,606	YES	YES	Alpha-lattice
Wheat_3	1403	78,606	YES	YES	Alpha-lattice
Wheat_4	1388	78,606	YES	YES	Alpha-lattice
Wheat_5	1398	78,606	YES	YES	Alpha-lattice
Wheat_6	1277	78,606	YES	YES	Alpha-lattice
EYT_1	776	2038	YES	YES	Alpha-lattice
EYT_2	775	2038	YES	YES	Alpha-lattice
EYT_3	964	2038	YES	YES	Alpha-lattice
Disease	438	11,617	YES	YES	RCBD

**Table 2:** Outlier detection methods that combine *p*-values to calculate overall significance, where: $p_{k}\;$ denotes the statistical significance value from kth methods for an individual or genotype; K: different methods for which *p*-values can be combined; df: degrees of freedom; N: normal distribution; t: central t-distribution; χ^2^: central Chi-square distribution.

Methods	Authors	Test Statistics	Transformed Variable	$\mathrm{Dist}.\;\mathrm{Under}\;H_{0}$
Inverse Chi-Square (Invchi)	Won, et al. (2009) [31].	$L=\sum_{k=1}^{K}Z_{k}$	$Z_{k}=-2logp_{k}$	$\chi_{2K}^{2}$
Logit	Mudholkar and George (1979) [32].	$S=\sum_{k=1}^{K}S_{k}$	$S_{k}=log\left(\frac{p_{k}}{1-p_{k}}\right)$	$t_{5K+4}$
Meanp	Sutton, et al. (2000) [33]	$W=(0.5-\bar{p})\sqrt{12K}$	$\bar{p}=\frac{\sum_{k=1}^{K}p_{k}}{K}$	N(0,1)
SumZ	Stouffer, et al. (1949) [34]	$Z=\frac{\sum_{k=1}^{K}w_{k}z(p_{k})}{\sqrt{\sum_{k=1}^{K}w_{k}^{2}}}$	NA	N(0,1)

**Figure A1:**
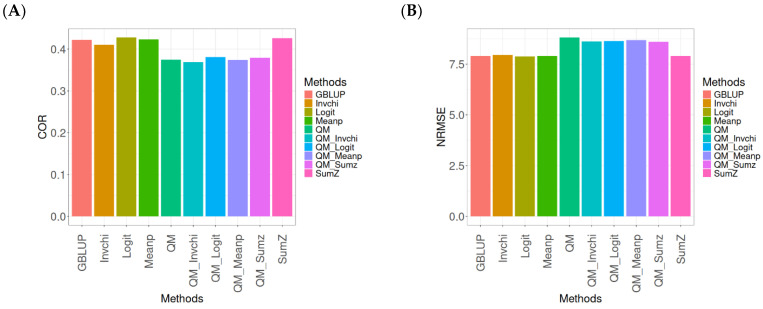
Comparative performance of genomic prediction methods in terms of Pearson’s correlation (COR) (**A**) and the normalized root mean square error (NRMSE) (**B**) for the Maize dataset, using quantile mapping.

**Figure A2:**
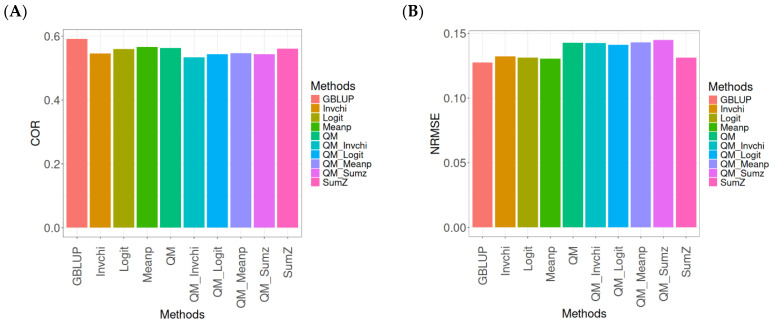
Comparative performance of genomic prediction methods in terms of Pearson’s correlation (COR) (**A**) and the normalized root mean square error (NRMSE) (**B**) for the Japonica dataset, using quantile mapping.

**Figure A3:**
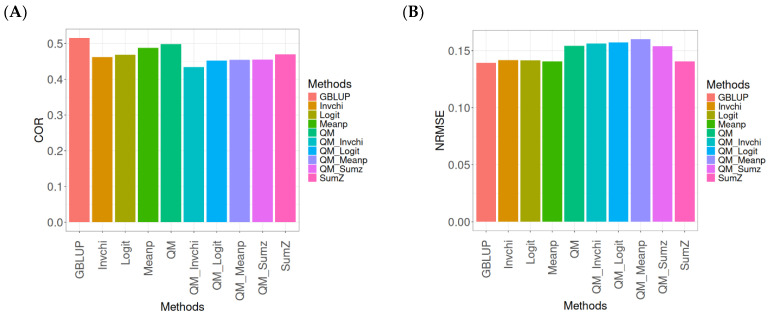
Comparative performance of genomic prediction methods in terms of Pearson’s correlation (COR) (**A**) and the normalized root mean square error (NRMSE) (**B**) for the Indica dataset, using quantile mapping.

**Figure A4:**
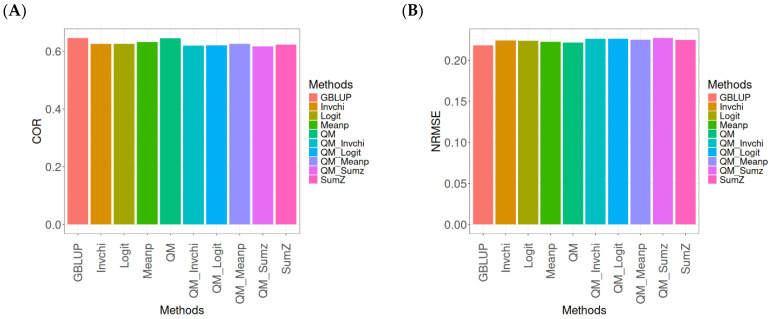
Comparative performance of genomic prediction methods in terms of Pearson’s correlation (COR) (**A**) and the normalized root mean square error (NRMSE) (**B**) for the Groundnut dataset, using quantile mapping.

**Figure A5:**
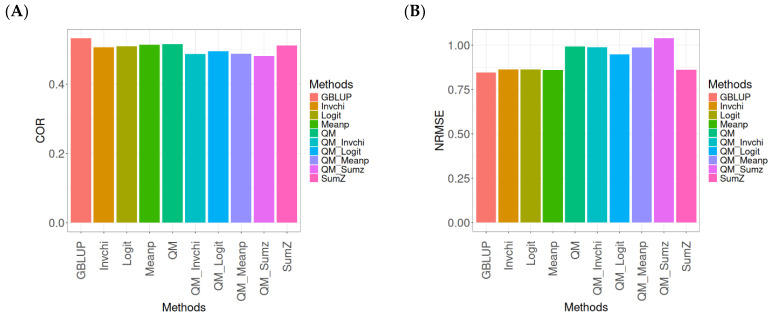
Comparative performance of genomic prediction methods in terms of Pearson’s correlation (COR) (**A**) and the normalized root mean square error (NRMSE) (**B**) for the EYT_2 dataset, using quantile mapping.

**Figure A6:**
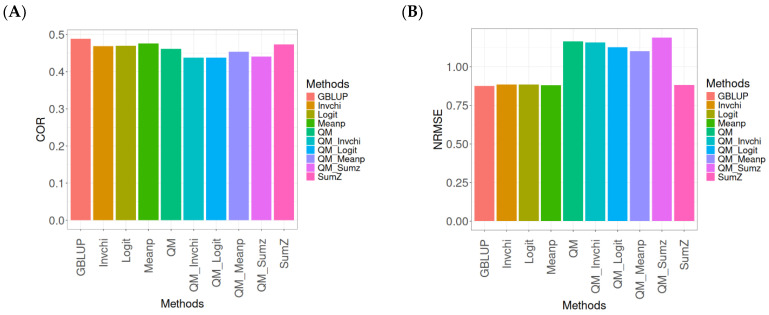
Comparative performance of genomic prediction methods in terms of Pearson’s correlation (COR) (**A**) and the normalized root mean square error (NRMSE) (**B**) for the EYT_3 dataset, using quantile mapping.

**Figure A7:**
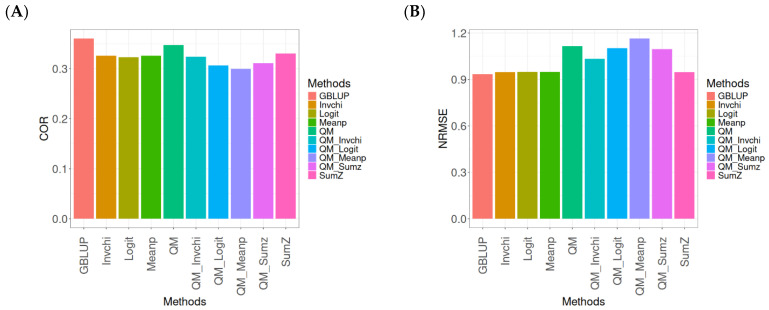
Comparative performance of genomic prediction methods in terms of Pearson’s correlation (COR) (**A**) and the Normalized Root Mean Square Error (NRMSE) (**B**) for the Wheat_2 dataset, using quantile mapping.

**Figure A8:**
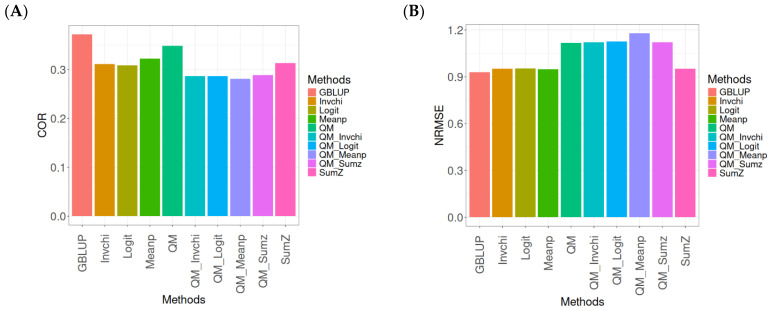
Comparative performance of genomic prediction methods in terms of Pearson’s correlation (COR) (**A**) and the normalized root mean square error (NRMSE) (**B**) for the Wheat_3 dataset, using quantile mapping.

**Figure A9:**
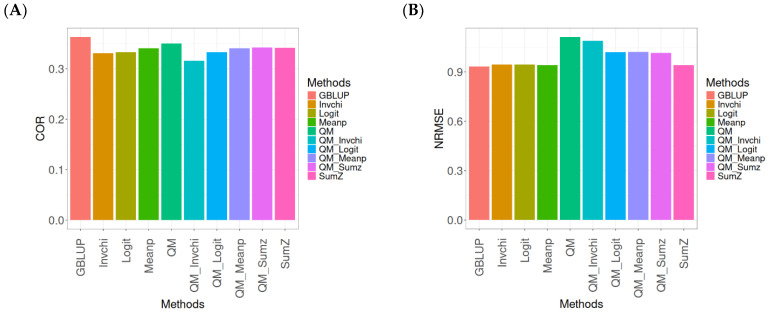
Comparative performance of genomic prediction methods in terms of Pearson’s correlation (COR) (**A**) and the normalized root mean square error (NRMSE) (**B**) for the Wheat_4 dataset, using quantile mapping.

**Figure A10:**
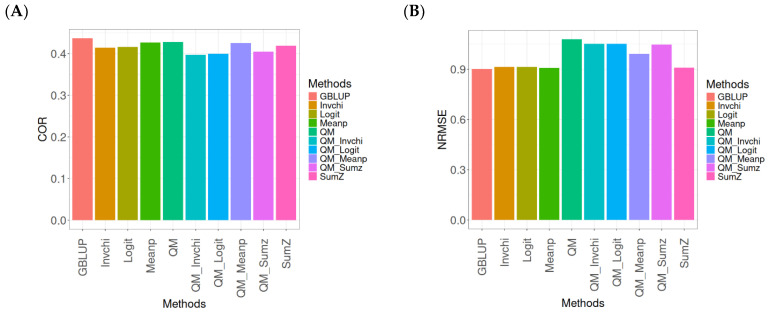
Comparative performance of genomic prediction methods in terms of Pearson’s correlation (COR) (**A**) and the normalized root mean square error (NRMSE) (**B**) for the Wheat_5 dataset, using quantile mapping.

**Figure A11:**
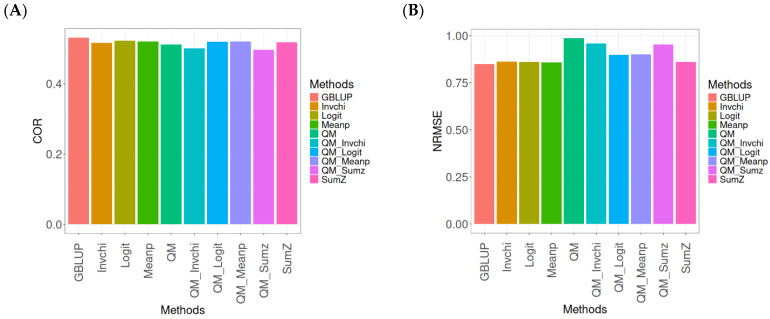
Comparative performance of genomic prediction methods in terms of Pearson’s correlation (COR) (**A**) and the normalized root mean square error (NRMSE) (**B**) for the Wheat_6 dataset, using quantile mapping.

## Data Availability

The genomic and phenotypic data used in this study are available at the following link. https://github.com/osval78/Refaning_Penalized_Regression, accessed on 8 January 2025.

## Abbreviations

GS	Genomic selection
GBLUP	Genomic best linear unbiased predictor
QM	Quantile mapping
CDF	Cumulative density function
COR	Correlation
NRMSE	Normalized root mean square error

## Appendix A

Results for datasets Disease (Table A1), EYT_1 (Table A2), Wheat_1 (Table A3), and across datasets (Table A4).

**Table A1:** Performance comparison of genomic prediction methods in terms of Pearson’s correlation (COR) (A) and the normalized root mean square error (NRMSE) (B) for the Disease dataset, using quantile mapping.

Metric	Method	Min	Mean	Median	Max
COR	GBLUP	0.0976	0.1766	0.1977	0.2344
COR	Invchi	0.0544	0.1552	0.1820	0.2293
COR	Logit	0.0506	0.1559	0.1829	0.2344
COR	Meanp	0.1008	0.1728	0.1755	0.2421
COR	QM	0.1018	0.1751	0.1882	0.2351
COR	QM_Invchi	0.0511	0.1530	0.1844	0.2234
COR	QM_Logit	0.0486	0.1528	0.1841	0.2258
COR	QM_Meanp	0.0842	0.1661	0.1735	0.2407
COR	QM_Sumz	0.0699	0.1586	0.1855	0.2204
COR	SumZ	0.0681	0.1630	0.1917	0.2291
COR_SE	GBLUP	0.0169	0.0234	0.0242	0.0290
COR_SE	Invchi	0.0276	0.0309	0.0323	0.0327
COR_SE	Logit	0.0221	0.0317	0.0341	0.0388
COR_SE	Meanp	0.0247	0.0272	0.0252	0.0318
COR_SE	QM	0.0206	0.0247	0.0228	0.0307
COR_SE	QM_Invchi	0.0303	0.0326	0.0323	0.0352
COR_SE	QM_Logit	0.0227	0.0320	0.0328	0.0407
COR_SE	QM_Meanp	0.0241	0.0267	0.0241	0.0318
COR_SE	QM_Sumz	0.0201	0.0282	0.0306	0.0340
COR_SE	SumZ	0.0200	0.0274	0.0295	0.0327
NRMSE	GBLUP	0.4055	0.4313	0.4127	0.4757
NRMSE	Invchi	0.4066	0.4346	0.4136	0.4837
NRMSE	Logit	0.4060	0.4345	0.4135	0.4840
NRMSE	Meanp	0.4044	0.4318	0.4149	0.4761
NRMSE	QM	0.4670	0.5234	0.5028	0.6005
NRMSE	QM_Invchi	0.4464	0.4986	0.4728	0.5767
NRMSE	QM_Logit	0.4439	0.4984	0.4750	0.5762
NRMSE	QM_Meanp	0.4464	0.5072	0.4954	0.5798
NRMSE	QM_Sumz	0.4463	0.4987	0.4741	0.5756
NRMSE	SumZ	0.4063	0.4337	0.4127	0.4820
NRMSE_SE	GBLUP	0.0032	0.0063	0.0065	0.0092
NRMSE_SE	Invchi	0.0039	0.0066	0.0064	0.0095
NRMSE_SE	Logit	0.0043	0.0065	0.0058	0.0093
NRMSE_SE	Meanp	0.0036	0.0062	0.0058	0.0093
NRMSE_SE	QM	0.0076	0.0091	0.0090	0.0107
NRMSE_SE	QM_Invchi	0.0077	0.0100	0.0105	0.0119
NRMSE_SE	QM_Logit	0.0069	0.0089	0.0099	0.0101
NRMSE_SE	QM_Meanp	0.0076	0.0102	0.0111	0.0118
NRMSE_SE	QM_Sumz	0.0059	0.0091	0.0098	0.0116
NRMSE_SE	SumZ	0.0043	0.0066	0.0062	0.0093

**Table A2:** Performance comparison of genomic prediction methods in terms of Pearson’s correlation (COR) (A) and the normalized root mean square error (NRMSE) (B) for the EYT_1 dataset, using quantile mapping.

Metric	Method	Min	Mean	Median	Max
COR	GBLUP	0.4282	0.4659	0.4727	0.4901
COR	Invchi	0.4037	0.4417	0.4445	0.4741
COR	Logit	0.3937	0.4414	0.4477	0.4765
COR	Meanp	0.4028	0.4480	0.4570	0.4753
COR	QM	0.3542	0.4429	0.4633	0.4908
COR	QM_Invchi	0.3588	0.4193	0.4283	0.4617
COR	QM_Logit	0.3715	0.4257	0.4306	0.4698
COR	QM_Meanp	0.3393	0.4273	0.4477	0.4746
COR	QM_Sumz	0.3794	0.4270	0.4292	0.4702
COR	SumZ	0.3963	0.4389	0.4438	0.4717
COR_SE	GBLUP	0.0096	0.0164	0.0151	0.0256
COR_SE	Invchi	0.0162	0.0196	0.0178	0.0264
COR_SE	Logit	0.0153	0.0184	0.0181	0.0220
COR_SE	Meanp	0.0126	0.0179	0.0167	0.0257
COR_SE	QM	0.0109	0.0234	0.0197	0.0432
COR_SE	QM_Invchi	0.0193	0.0279	0.0291	0.0339
COR_SE	QM_Logit	0.0156	0.0226	0.0233	0.0284
COR_SE	QM_Meanp	0.0122	0.0226	0.0222	0.0338
COR_SE	QM_Sumz	0.0120	0.0225	0.0236	0.0308
COR_SE	SumZ	0.0126	0.0190	0.0191	0.0252
NRMSE	GBLUP	0.0349	0.0450	0.0448	0.0552
NRMSE	Invchi	0.0355	0.0455	0.0455	0.0557
NRMSE	Logit	0.0354	0.0456	0.0456	0.0556
NRMSE	Meanp	0.0353	0.0454	0.0453	0.0558
NRMSE	QM	0.0386	0.0545	0.0575	0.0646
NRMSE	QM_Invchi	0.0428	0.0533	0.0538	0.0628
NRMSE	QM_Logit	0.0377	0.0519	0.0536	0.0629
NRMSE	QM_Meanp	0.0376	0.0534	0.0571	0.0617
NRMSE	QM_Sumz	0.0380	0.0512	0.0522	0.0626
NRMSE	SumZ	0.0355	0.0456	0.0456	0.0558
NRMSE_SE	GBLUP	0.0004	0.0006	0.0006	0.0010
NRMSE_SE	Invchi	0.0005	0.0008	0.0008	0.0011
NRMSE_SE	Logit	0.0006	0.0008	0.0007	0.0012
NRMSE_SE	Meanp	0.0006	0.0008	0.0008	0.0010
NRMSE_SE	QM	0.0003	0.0031	0.0023	0.0076
NRMSE_SE	QM_Invchi	0.0010	0.0040	0.0043	0.0064
NRMSE_SE	QM_Logit	0.0006	0.0033	0.0038	0.0049
NRMSE_SE	QM_Meanp	0.0006	0.0032	0.0024	0.0074
NRMSE_SE	QM_Sumz	0.0006	0.0026	0.0024	0.0049
NRMSE_SE	SumZ	0.0006	0.0008	0.0007	0.0010

**Table A3:** Performance comparison of genomic prediction methods in terms of Pearson’s correlation (COR) (A) and the normalized root mean square error (NRMSE) (B) for the Wheat_1 dataset, using quantile mapping.

Metric	Method	Min	Mean	Median	Max
COR	GBLUP	0.4682	0.4682	0.4682	0.4682
COR	Invchi	0.4314	0.4314	0.4314	0.4314
COR	Logit	0.4387	0.4387	0.4387	0.4387
COR	Meanp	0.4406	0.4406	0.4406	0.4406
COR	QM	0.4508	0.4508	0.4508	0.4508
COR	QM_Invchi	0.4256	0.4256	0.4256	0.4256
COR	QM_Logit	0.4187	0.4187	0.4187	0.4187
COR	QM_Meanp	0.4299	0.4299	0.4299	0.4299
COR	QM_Sumz	0.4214	0.4214	0.4214	0.4214
COR	SumZ	0.4400	0.4400	0.4400	0.4400
COR_SE	GBLUP	0.0149	0.0149	0.0149	0.0149
COR_SE	Invchi	0.0116	0.0116	0.0116	0.0116
COR_SE	Logit	0.0123	0.0123	0.0123	0.0123
COR_SE	Meanp	0.0122	0.0122	0.0122	0.0122
COR_SE	QM	0.0175	0.0175	0.0175	0.0175
COR_SE	QM_Invchi	0.0161	0.0161	0.0161	0.0161
COR_SE	QM_Logit	0.0129	0.0129	0.0129	0.0129
COR_SE	QM_Meanp	0.0137	0.0137	0.0137	0.0137
COR_SE	QM_Sumz	0.0166	0.0166	0.0166	0.0166
COR_SE	SumZ	0.0127	0.0127	0.0127	0.0127
NRMSE	GBLUP	0.8870	0.8870	0.8870	0.8870
NRMSE	Invchi	0.9047	0.9047	0.9047	0.9047
NRMSE	Logit	0.9009	0.9009	0.9009	0.9009
NRMSE	Meanp	0.9015	0.9015	0.9015	0.9015
NRMSE	QM	1.0293	1.0293	1.0293	1.0293
NRMSE	QM_Invchi	0.9866	0.9866	0.9866	0.9866
NRMSE	QM_Logit	1.0148	1.0148	1.0148	1.0148
NRMSE	QM_Meanp	0.9895	0.9895	0.9895	0.9895
NRMSE	QM_Sumz	1.0238	1.0238	1.0238	1.0238
NRMSE	SumZ	0.9016	0.9016	0.9016	0.9016
NRMSE_SE	GBLUP	0.0092	0.0092	0.0092	0.0092
NRMSE_SE	Invchi	0.0053	0.0053	0.0053	0.0053
NRMSE_SE	Logit	0.0060	0.0060	0.0060	0.0060
NRMSE_SE	Meanp	0.0055	0.0055	0.0055	0.0055
NRMSE_SE	QM	0.0436	0.0436	0.0436	0.0436
NRMSE_SE	QM_Invchi	0.0367	0.0367	0.0367	0.0367
NRMSE_SE	QM_Logit	0.0363	0.0363	0.0363	0.0363
NRMSE_SE	QM_Meanp	0.0367	0.0367	0.0367	0.0367
NRMSE_SE	QM_Sumz	0.0461	0.0461	0.0461	0.0461
NRMSE_SE	SumZ	0.0059	0.0059	0.0059	0.0059

**Table A4:** Performance comparison of genomic prediction methods in terms of Pearson’s correlation (COR) (A) and the normalized root mean square error (NRMSE) (B) across the datasets, using quantile mapping.

Metric	Method	Min	Mean	Median	Max
COR	GBLUP	0.0976	0.4834	0.4937	0.6941
COR	Invchi	0.0544	0.4533	0.4682	0.6667
COR	Logit	0.0506	0.4569	0.4728	0.6676
COR	Meanp	0.1008	0.4649	0.4751	0.6739
COR	QM	0.1018	0.4659	0.4805	0.6978
COR	QM_Invchi	0.0511	0.4355	0.4406	0.6653
COR	QM_Logit	0.0486	0.4412	0.4588	0.6668
COR	QM_Meanp	0.0842	0.4458	0.4593	0.6751
COR	QM_Sumz	0.0699	0.4405	0.4645	0.6633
COR	SumZ	0.0681	0.4584	0.4709	0.6662
COR_SE	GBLUP	0.0092	0.0200	0.0179	0.0503
COR_SE	Invchi	0.0107	0.0228	0.0197	0.0659
COR_SE	Logit	0.0073	0.0223	0.0199	0.0657
COR_SE	Meanp	0.0093	0.0222	0.0199	0.0605
COR_SE	QM	0.0109	0.0245	0.0225	0.0507
COR_SE	QM_Invchi	0.0140	0.0278	0.0246	0.0570
COR_SE	QM_Logit	0.0096	0.0255	0.0206	0.0668
COR_SE	QM_Meanp	0.0088	0.0265	0.0247	0.0550
COR_SE	QM_Sumz	0.0120	0.0256	0.0217	0.0502
COR_SE	SumZ	0.0097	0.0219	0.0191	0.0577
NRMSE	GBLUP	0.0297	0.6954	0.4210	7.9058
NRMSE	Invchi	0.0305	0.7043	0.4254	7.9443
NRMSE	Logit	0.0304	0.7019	0.4249	7.8848
NRMSE	Meanp	0.0300	0.7003	0.4236	7.8912
NRMSE	QM	0.0361	0.8160	0.4860	8.8085
NRMSE	QM_Invchi	0.0312	0.8018	0.4719	8.6206
NRMSE	QM_Logit	0.0377	0.7928	0.4765	8.6409
NRMSE	QM_Meanp	0.0376	0.7976	0.4829	8.6800
NRMSE	QM_Sumz	0.0380	0.8110	0.4701	8.6088
NRMSE	SumZ	0.0300	0.7019	0.4221	7.9065
NRMSE_SE	GBLUP	0.0004	0.0885	0.0064	2.7860
NRMSE_SE	Invchi	0.0005	0.0885	0.0063	2.7937
NRMSE_SE	Logit	0.0006	0.0882	0.0059	2.7865
NRMSE_SE	Meanp	0.0006	0.0878	0.0058	2.7730
NRMSE_SE	QM	0.0003	0.1316	0.0107	3.1423
NRMSE_SE	QM_Invchi	0.0010	0.1282	0.0104	3.0366
NRMSE_SE	QM_Logit	0.0006	0.1194	0.0102	3.0848
NRMSE_SE	QM_Meanp	0.0006	0.1295	0.0112	3.0857
NRMSE_SE	QM_Sumz	0.0006	0.1320	0.0124	3.0495
NRMSE_SE	SumZ	0.0006	0.0887	0.0063	2.8039

## Appendix B

Figures for datasets Maize (Figure A1), Japonica (Figure A2), Indica (Figure A3), Groundnut (Figure A4), EYT_2 (Figure A5), EYT_3 (Figure A6), Wheat_2 (Figure A7), Wheat_3 (Figure A8), Wheat_4 (Figure A9), Wheat_5 (Figure A10), and Wheat_6 (Figure A11).

## Appendix C

Table of results for datasets Maize, Japonica, Indica, Groundnut, EYT_2, EYT_3, Wheat_2, Wheat_3, Wheat_4, Wheat_5, and Wheat_6.

**Table A5:** Comparative performance of genomic prediction models in terms of Pearson’s correlation (COR and COR standard error COR_SE) and the normalized root mean square error (NRMSE and NRMSE standard error, NRMSE_SE) for Maize, Japonica, Indica, Groundnut, EYT_2, EYT_3, Wheat_2, Wheat_3, Wheat_4, Wheat_5, and Wheat_6 datasets.

Data	Metric	Method	Min	Mean	Median	Max
Maize	COR	GBLUP	0.4225	0.4225	0.4225	0.4225
Maize	COR	Invchi	0.4106	0.4106	0.4106	0.4106
Maize	COR	Logit	0.4276	0.4276	0.4276	0.4276
Maize	COR	Meanp	0.4235	0.4235	0.4235	0.4235
Maize	COR	QM	0.3748	0.3748	0.3748	0.3748
Maize	COR	QM_Invchi	0.3691	0.3691	0.3691	0.3691
Maize	COR	QM_Logit	0.3809	0.3809	0.3809	0.3809
Maize	COR	QM_Meanp	0.3741	0.3741	0.3741	0.3741
Maize	COR	QM_Sumz	0.3792	0.3792	0.3792	0.3792
Maize	COR	SumZ	0.4264	0.4264	0.4264	0.4264
Maize	COR_SE	GBLUP	0.0180	0.0180	0.0180	0.0180
Maize	COR_SE	Invchi	0.0173	0.0173	0.0173	0.0173
Maize	COR_SE	Logit	0.0176	0.0176	0.0176	0.0176
Maize	COR_SE	Meanp	0.0174	0.0174	0.0174	0.0174
Maize	COR_SE	QM	0.0229	0.0229	0.0229	0.0229
Maize	COR_SE	QM_Invchi	0.0237	0.0237	0.0237	0.0237
Maize	COR_SE	QM_Logit	0.0201	0.0201	0.0201	0.0201
Maize	COR_SE	QM_Meanp	0.0218	0.0218	0.0218	0.0218
Maize	COR_SE	QM_Sumz	0.0206	0.0206	0.0206	0.0206
Maize	COR_SE	SumZ	0.0175	0.0175	0.0175	0.0175
Maize	NRMSE	GBLUP	7.9058	7.9058	7.9058	7.9058
Maize	NRMSE	Invchi	7.9443	7.9443	7.9443	7.9443
Maize	NRMSE	Logit	7.8848	7.8848	7.8848	7.8848
Maize	NRMSE	Meanp	7.8912	7.8912	7.8912	7.8912
Maize	NRMSE	QM	8.8085	8.8085	8.8085	8.8085
Maize	NRMSE	QM_Invchi	8.6206	8.6206	8.6206	8.6206
Maize	NRMSE	QM_Logit	8.6409	8.6409	8.6409	8.6409
Maize	NRMSE	QM_Meanp	8.6800	8.6800	8.6800	8.6800
Maize	NRMSE	QM_Sumz	8.6088	8.6088	8.6088	8.6088
Maize	NRMSE	SumZ	7.9065	7.9065	7.9065	7.9065
Maize	NRMSE_SE	GBLUP	2.7860	2.7860	2.7860	2.7860
Maize	NRMSE_SE	Invchi	2.7937	2.7937	2.7937	2.7937
Maize	NRMSE_SE	Logit	2.7865	2.7865	2.7865	2.7865
Maize	NRMSE_SE	Meanp	2.7730	2.7730	2.7730	2.7730
Maize	NRMSE_SE	QM	3.1423	3.1423	3.1423	3.1423
Maize	NRMSE_SE	QM_Invchi	3.0366	3.0366	3.0366	3.0366
Maize	NRMSE_SE	QM_Logit	3.0848	3.0848	3.0848	3.0848
Maize	NRMSE_SE	QM_Meanp	3.0857	3.0857	3.0857	3.0857
Maize	NRMSE_SE	QM_Sumz	3.0495	3.0495	3.0495	3.0495
Maize	NRMSE_SE	SumZ	2.8039	2.8039	2.8039	2.8039
Japonica	COR	GBLUP	0.5681	0.5914	0.5803	0.6366
Japonica	COR	Invchi	0.5266	0.5461	0.5412	0.5753
Japonica	COR	Logit	0.5372	0.5602	0.5602	0.5831
Japonica	COR	Meanp	0.5478	0.5662	0.5637	0.5896
Japonica	COR	QM	0.5246	0.5633	0.5691	0.5905
Japonica	COR	QM_Invchi	0.4955	0.5333	0.5320	0.5737
Japonica	COR	QM_Logit	0.5171	0.5430	0.5364	0.5821
Japonica	COR	QM_Meanp	0.5164	0.5473	0.5421	0.5884
Japonica	COR	QM_Sumz	0.5068	0.5430	0.5378	0.5895
Japonica	COR	SumZ	0.5376	0.5609	0.5580	0.5901
Japonica	COR_SE	GBLUP	0.0182	0.0233	0.0198	0.0352
Japonica	COR_SE	Invchi	0.0167	0.0279	0.0262	0.0426
Japonica	COR_SE	Logit	0.0201	0.0285	0.0272	0.0395
Japonica	COR_SE	Meanp	0.0218	0.0297	0.0270	0.0428
Japonica	COR_SE	QM	0.0187	0.0337	0.0328	0.0507
Japonica	COR_SE	QM_Invchi	0.0178	0.0278	0.0272	0.0390
Japonica	COR_SE	QM_Logit	0.0197	0.0370	0.0307	0.0668
Japonica	COR_SE	QM_Meanp	0.0217	0.0329	0.0319	0.0460
Japonica	COR_SE	QM_Sumz	0.0173	0.0301	0.0270	0.0490
Japonica	COR_SE	SumZ	0.0175	0.0287	0.0276	0.0422
Japonica	NRMSE	GBLUP	0.0297	0.1274	0.0524	0.3752
Japonica	NRMSE	Invchi	0.0305	0.1322	0.0546	0.3891
Japonica	NRMSE	Logit	0.0304	0.1313	0.0540	0.3869
Japonica	NRMSE	Meanp	0.0300	0.1303	0.0538	0.3837
Japonica	NRMSE	QM	0.0413	0.1427	0.0598	0.4100
Japonica	NRMSE	QM_Invchi	0.0312	0.1424	0.0606	0.4171
Japonica	NRMSE	QM_Logit	0.0409	0.1413	0.0558	0.4126
Japonica	NRMSE	QM_Meanp	0.0487	0.1430	0.0558	0.4117
Japonica	NRMSE	QM_Sumz	0.0392	0.1448	0.0622	0.4157
Japonica	NRMSE	SumZ	0.0300	0.1312	0.0541	0.3866
Japonica	NRMSE_SE	GBLUP	0.0010	0.0045	0.0030	0.0110
Japonica	NRMSE_SE	Invchi	0.0009	0.0049	0.0038	0.0110
Japonica	NRMSE_SE	Logit	0.0010	0.0047	0.0037	0.0106
Japonica	NRMSE_SE	Meanp	0.0009	0.0049	0.0038	0.0110
Japonica	NRMSE_SE	QM	0.0018	0.0078	0.0093	0.0107
Japonica	NRMSE_SE	QM_Invchi	0.0011	0.0054	0.0051	0.0102
Japonica	NRMSE_SE	QM_Logit	0.0016	0.0066	0.0072	0.0103
Japonica	NRMSE_SE	QM_Meanp	0.0017	0.0073	0.0078	0.0118
Japonica	NRMSE_SE	QM_Sumz	0.0018	0.0081	0.0086	0.0135
Japonica	NRMSE_SE	SumZ	0.0008	0.0047	0.0038	0.0106
Indica	COR	GBLUP	0.3510	0.5151	0.5283	0.6530
Indica	COR	Invchi	0.2863	0.4622	0.4592	0.6439
Indica	COR	Logit	0.2919	0.4682	0.4644	0.6523
Indica	COR	Meanp	0.3178	0.4874	0.4917	0.6484
Indica	COR	QM	0.3509	0.4982	0.5026	0.6367
Indica	COR	QM_Invchi	0.2783	0.4346	0.4326	0.5947
Indica	COR	QM_Logit	0.2903	0.4524	0.4549	0.6097
Indica	COR	QM_Meanp	0.3266	0.4544	0.4443	0.6025
Indica	COR	QM_Sumz	0.2847	0.4552	0.4584	0.6192
Indica	COR	SumZ	0.3072	0.4696	0.4650	0.6413
Indica	COR_SE	GBLUP	0.0268	0.0360	0.0334	0.0503
Indica	COR_SE	Invchi	0.0212	0.0423	0.0411	0.0659
Indica	COR_SE	Logit	0.0233	0.0392	0.0339	0.0657
Indica	COR_SE	Meanp	0.0284	0.0403	0.0361	0.0605
Indica	COR_SE	QM	0.0230	0.0359	0.0361	0.0486
Indica	COR_SE	QM_Invchi	0.0433	0.0493	0.0484	0.0570
Indica	COR_SE	QM_Logit	0.0197	0.0389	0.0386	0.0586
Indica	COR_SE	QM_Meanp	0.0352	0.0456	0.0462	0.0550
Indica	COR_SE	QM_Sumz	0.0225	0.0372	0.0381	0.0502
Indica	COR_SE	SumZ	0.0233	0.0376	0.0346	0.0577
Indica	NRMSE	GBLUP	0.0335	0.1393	0.0473	0.4293
Indica	NRMSE	Invchi	0.0366	0.1419	0.0468	0.4372
Indica	NRMSE	Logit	0.0365	0.1415	0.0465	0.4363
Indica	NRMSE	Meanp	0.0353	0.1405	0.0471	0.4324
Indica	NRMSE	QM	0.0361	0.1544	0.0561	0.4691
Indica	NRMSE	QM_Invchi	0.0392	0.1562	0.0573	0.4709
Indica	NRMSE	QM_Logit	0.0425	0.1571	0.0540	0.4780
Indica	NRMSE	QM_Meanp	0.0527	0.1601	0.0586	0.4704
Indica	NRMSE	QM_Sumz	0.0427	0.1538	0.0533	0.4661
Indica	NRMSE	SumZ	0.0369	0.1405	0.0468	0.4315
Indica	NRMSE_SE	GBLUP	0.0015	0.0070	0.0019	0.0228
Indica	NRMSE_SE	Invchi	0.0015	0.0074	0.0019	0.0241
Indica	NRMSE_SE	Logit	0.0017	0.0075	0.0018	0.0248
Indica	NRMSE_SE	Meanp	0.0017	0.0075	0.0019	0.0247
Indica	NRMSE_SE	QM	0.0014	0.0081	0.0050	0.0209
Indica	NRMSE_SE	QM_Invchi	0.0018	0.0096	0.0084	0.0198
Indica	NRMSE_SE	QM_Logit	0.0014	0.0098	0.0081	0.0217
Indica	NRMSE_SE	QM_Meanp	0.0081	0.0122	0.0099	0.0210
Indica	NRMSE_SE	QM_Sumz	0.0014	0.0087	0.0069	0.0196
Indica	NRMSE_SE	SumZ	0.0015	0.0073	0.0019	0.0237
Groundnut	COR	GBLUP	0.5928	0.6457	0.6480	0.6941
Groundnut	COR	Invchi	0.5673	0.6257	0.6345	0.6667
Groundnut	COR	Logit	0.5723	0.6257	0.6315	0.6676
Groundnut	COR	Meanp	0.5828	0.6311	0.6339	0.6739
Groundnut	COR	QM	0.5910	0.6445	0.6446	0.6978
Groundnut	COR	QM_Invchi	0.5588	0.6201	0.6282	0.6653
Groundnut	COR	QM_Logit	0.5653	0.6207	0.6253	0.6668
Groundnut	COR	QM_Meanp	0.5764	0.6261	0.6265	0.6751
Groundnut	COR	QM_Sumz	0.5595	0.6178	0.6242	0.6633
Groundnut	COR	SumZ	0.5664	0.6230	0.6298	0.6662
Groundnut	COR_SE	GBLUP	0.0117	0.0162	0.0168	0.0193
Groundnut	COR_SE	Invchi	0.0144	0.0182	0.0184	0.0217
Groundnut	COR_SE	Logit	0.0162	0.0187	0.0189	0.0206
Groundnut	COR_SE	Meanp	0.0174	0.0186	0.0178	0.0214
Groundnut	COR_SE	QM	0.0117	0.0161	0.0169	0.0187
Groundnut	COR_SE	QM_Invchi	0.0143	0.0190	0.0194	0.0232
Groundnut	COR_SE	QM_Logit	0.0157	0.0187	0.0193	0.0207
Groundnut	COR_SE	QM_Meanp	0.0177	0.0193	0.0191	0.0212
Groundnut	COR_SE	QM_Sumz	0.0172	0.0189	0.0187	0.0210
Groundnut	COR_SE	SumZ	0.0164	0.0188	0.0186	0.0215
Groundnut	NRMSE	GBLUP	0.1857	0.2185	0.2098	0.2688
Groundnut	NRMSE	Invchi	0.1932	0.2243	0.2140	0.2760
Groundnut	NRMSE	Logit	0.1927	0.2239	0.2144	0.2741
Groundnut	NRMSE	Meanp	0.1913	0.2225	0.2118	0.2749
Groundnut	NRMSE	QM	0.1852	0.2219	0.2151	0.2720
Groundnut	NRMSE	QM_Invchi	0.1930	0.2264	0.2170	0.2787
Groundnut	NRMSE	QM_Logit	0.1924	0.2264	0.2181	0.2772
Groundnut	NRMSE	QM_Meanp	0.1901	0.2249	0.2153	0.2790
Groundnut	NRMSE	QM_Sumz	0.1924	0.2271	0.2178	0.2803
Groundnut	NRMSE	SumZ	0.1927	0.2246	0.2148	0.2764
Groundnut	NRMSE_SE	GBLUP	0.0042	0.0064	0.0066	0.0083
Groundnut	NRMSE_SE	Invchi	0.0041	0.0070	0.0072	0.0095
Groundnut	NRMSE_SE	Logit	0.0043	0.0068	0.0073	0.0083
Groundnut	NRMSE_SE	Meanp	0.0043	0.0069	0.0072	0.0089
Groundnut	NRMSE_SE	QM	0.0045	0.0065	0.0063	0.0090
Groundnut	NRMSE_SE	QM_Invchi	0.0043	0.0067	0.0064	0.0097
Groundnut	NRMSE_SE	QM_Logit	0.0049	0.0065	0.0063	0.0083
Groundnut	NRMSE_SE	QM_Meanp	0.0047	0.0068	0.0067	0.0091
Groundnut	NRMSE_SE	QM_Sumz	0.0049	0.0066	0.0062	0.0090
Groundnut	NRMSE_SE	SumZ	0.0043	0.0068	0.0071	0.0088
EYT_2	COR	GBLUP	0.4493	0.5320	0.5296	0.6196
EYT_2	COR	Invchi	0.4341	0.5066	0.5039	0.5846
EYT_2	COR	Logit	0.4358	0.5092	0.5082	0.5845
EYT_2	COR	Meanp	0.4385	0.5133	0.5092	0.5961
EYT_2	COR	QM	0.4135	0.5154	0.5163	0.6157
EYT_2	COR	QM_Invchi	0.3956	0.4867	0.4893	0.5726
EYT_2	COR	QM_Logit	0.3959	0.4946	0.5063	0.5701
EYT_2	COR	QM_Meanp	0.4177	0.4881	0.4915	0.5516
EYT_2	COR	QM_Sumz	0.3730	0.4807	0.4848	0.5805
EYT_2	COR	SumZ	0.4373	0.5113	0.5086	0.5909
EYT_2	COR_SE	GBLUP	0.0127	0.0154	0.0147	0.0193
EYT_2	COR_SE	Invchi	0.0146	0.0194	0.0202	0.0228
EYT_2	COR_SE	Logit	0.0160	0.0185	0.0175	0.0230
EYT_2	COR_SE	Meanp	0.0153	0.0183	0.0180	0.0219
EYT_2	COR_SE	QM	0.0134	0.0219	0.0223	0.0297
EYT_2	COR_SE	QM_Invchi	0.0203	0.0255	0.0249	0.0319
EYT_2	COR_SE	QM_Logit	0.0162	0.0209	0.0180	0.0313
EYT_2	COR_SE	QM_Meanp	0.0159	0.0259	0.0261	0.0352
EYT_2	COR_SE	QM_Sumz	0.0202	0.0247	0.0249	0.0287
EYT_2	COR_SE	SumZ	0.0153	0.0188	0.0185	0.0231
EYT_2	NRMSE	GBLUP	0.7866	0.8463	0.8508	0.8970
EYT_2	NRMSE	Invchi	0.8159	0.8640	0.8674	0.9054
EYT_2	NRMSE	Logit	0.8164	0.8628	0.8653	0.9043
EYT_2	NRMSE	Meanp	0.8084	0.8602	0.8649	0.9027
EYT_2	NRMSE	QM	0.8378	0.9930	0.9979	1.1382
EYT_2	NRMSE	QM_Invchi	0.8768	0.9898	0.9869	1.1086
EYT_2	NRMSE	QM_Logit	0.8777	0.9479	0.9020	1.1098
EYT_2	NRMSE	QM_Meanp	0.9213	0.9863	0.9926	1.0385
EYT_2	NRMSE	QM_Sumz	0.8717	1.0384	1.0440	1.1939
EYT_2	NRMSE	SumZ	0.8117	0.8619	0.8664	0.9032
EYT_2	NRMSE_SE	GBLUP	0.0077	0.0100	0.0105	0.0114
EYT_2	NRMSE_SE	Invchi	0.0090	0.0109	0.0111	0.0124
EYT_2	NRMSE_SE	Logit	0.0092	0.0106	0.0104	0.0125
EYT_2	NRMSE_SE	Meanp	0.0097	0.0104	0.0101	0.0118
EYT_2	NRMSE_SE	QM	0.0115	0.0746	0.0611	0.1648
EYT_2	NRMSE_SE	QM_Invchi	0.0170	0.0824	0.0737	0.1652
EYT_2	NRMSE_SE	QM_Logit	0.0120	0.0429	0.0281	0.1036
EYT_2	NRMSE_SE	QM_Meanp	0.0149	0.0783	0.0894	0.1195
EYT_2	NRMSE_SE	QM_Sumz	0.0440	0.1105	0.1163	0.1654
EYT_2	NRMSE_SE	SumZ	0.0092	0.0108	0.0108	0.0124
EYT_3	COR	GBLUP	0.4760	0.4884	0.4881	0.5012
EYT_3	COR	Invchi	0.4604	0.4689	0.4661	0.4830
EYT_3	COR	Logit	0.4598	0.4695	0.4705	0.4771
EYT_3	COR	Meanp	0.4648	0.4755	0.4715	0.4944
EYT_3	COR	QM	0.4340	0.4618	0.4566	0.5002
EYT_3	COR	QM_Invchi	0.4104	0.4383	0.4307	0.4814
EYT_3	COR	QM_Logit	0.3839	0.4381	0.4474	0.4736
EYT_3	COR	QM_Meanp	0.4372	0.4535	0.4420	0.4930
EYT_3	COR	QM_Sumz	0.3968	0.4405	0.4486	0.4681
EYT_3	COR	SumZ	0.4667	0.4730	0.4710	0.4830
EYT_3	COR_SE	GBLUP	0.0137	0.0196	0.0203	0.0243
EYT_3	COR_SE	Invchi	0.0138	0.0185	0.0189	0.0224
EYT_3	COR_SE	Logit	0.0145	0.0184	0.0178	0.0234
EYT_3	COR_SE	Meanp	0.0134	0.0181	0.0179	0.0231
EYT_3	COR_SE	QM	0.0139	0.0244	0.0254	0.0329
EYT_3	COR_SE	QM_Invchi	0.0140	0.0274	0.0274	0.0406
EYT_3	COR_SE	QM_Logit	0.0147	0.0225	0.0200	0.0352
EYT_3	COR_SE	QM_Meanp	0.0133	0.0254	0.0279	0.0325
EYT_3	COR_SE	QM_Sumz	0.0158	0.0286	0.0291	0.0402
EYT_3	COR_SE	SumZ	0.0151	0.0179	0.0173	0.0218
EYT_3	NRMSE	GBLUP	0.8663	0.8758	0.8757	0.8854
EYT_3	NRMSE	Invchi	0.8750	0.8850	0.8866	0.8919
EYT_3	NRMSE	Logit	0.8787	0.8843	0.8842	0.8899
EYT_3	NRMSE	Meanp	0.8694	0.8809	0.8826	0.8892
EYT_3	NRMSE	QM	0.9549	1.1644	1.1869	1.3290
EYT_3	NRMSE	QM_Invchi	0.9226	1.1572	1.1758	1.3544
EYT_3	NRMSE	QM_Logit	0.9324	1.1272	1.0212	1.5341
EYT_3	NRMSE	QM_Meanp	0.9229	1.1000	1.1039	1.2693
EYT_3	NRMSE	QM_Sumz	0.9358	1.1875	1.1450	1.5243
EYT_3	NRMSE	SumZ	0.8755	0.8824	0.8835	0.8871
EYT_3	NRMSE_SE	GBLUP	0.0080	0.0116	0.0116	0.0154
EYT_3	NRMSE_SE	Invchi	0.0073	0.0100	0.0104	0.0117
EYT_3	NRMSE_SE	Logit	0.0076	0.0098	0.0094	0.0129
EYT_3	NRMSE_SE	Meanp	0.0070	0.0100	0.0102	0.0124
EYT_3	NRMSE_SE	QM	0.0128	0.1321	0.1500	0.2155
EYT_3	NRMSE_SE	QM_Invchi	0.0105	0.1374	0.1307	0.2777
EYT_3	NRMSE_SE	QM_Logit	0.0148	0.1040	0.0775	0.2461
EYT_3	NRMSE_SE	QM_Meanp	0.0113	0.1521	0.1362	0.3246
EYT_3	NRMSE_SE	QM_Sumz	0.0128	0.1383	0.1316	0.2773
EYT_3	NRMSE_SE	SumZ	0.0078	0.0097	0.0096	0.0117
Wheat_2	COR	GBLUP	0.3605	0.3605	0.3605	0.3605
Wheat_2	COR	Invchi	0.3258	0.3258	0.3258	0.3258
Wheat_2	COR	Logit	0.3236	0.3236	0.3236	0.3236
Wheat_2	COR	Meanp	0.3257	0.3257	0.3257	0.3257
Wheat_2	COR	QM	0.3476	0.3476	0.3476	0.3476
Wheat_2	COR	QM_Invchi	0.3241	0.3241	0.3241	0.3241
Wheat_2	COR	QM_Logit	0.3064	0.3064	0.3064	0.3064
Wheat_2	COR	QM_Meanp	0.2999	0.2999	0.2999	0.2999
Wheat_2	COR	QM_Sumz	0.3115	0.3115	0.3115	0.3115
Wheat_2	COR	SumZ	0.3303	0.3303	0.3303	0.3303
Wheat_2	COR_SE	GBLUP	0.0144	0.0144	0.0144	0.0144
Wheat_2	COR_SE	Invchi	0.0144	0.0144	0.0144	0.0144
Wheat_2	COR_SE	Logit	0.0153	0.0153	0.0153	0.0153
Wheat_2	COR_SE	Meanp	0.0162	0.0162	0.0162	0.0162
Wheat_2	COR_SE	QM	0.0216	0.0216	0.0216	0.0216
Wheat_2	COR_SE	QM_Invchi	0.0140	0.0140	0.0140	0.0140
Wheat_2	COR_SE	QM_Logit	0.0281	0.0281	0.0281	0.0281
Wheat_2	COR_SE	QM_Meanp	0.0291	0.0291	0.0291	0.0291
Wheat_2	COR_SE	QM_Sumz	0.0257	0.0257	0.0257	0.0257
Wheat_2	COR_SE	SumZ	0.0144	0.0144	0.0144	0.0144
Wheat_2	NRMSE	GBLUP	0.9340	0.9340	0.9340	0.9340
Wheat_2	NRMSE	Invchi	0.9477	0.9477	0.9477	0.9477
Wheat_2	NRMSE	Logit	0.9495	0.9495	0.9495	0.9495
Wheat_2	NRMSE	Meanp	0.9484	0.9484	0.9484	0.9484
Wheat_2	NRMSE	QM	1.1166	1.1166	1.1166	1.1166
Wheat_2	NRMSE	QM_Invchi	1.0345	1.0345	1.0345	1.0345
Wheat_2	NRMSE	QM_Logit	1.1027	1.1027	1.1027	1.1027
Wheat_2	NRMSE	QM_Meanp	1.1647	1.1647	1.1647	1.1647
Wheat_2	NRMSE	QM_Sumz	1.0955	1.0955	1.0955	1.0955
Wheat_2	NRMSE	SumZ	0.9470	0.9470	0.9470	0.9470
Wheat_2	NRMSE_SE	GBLUP	0.0053	0.0053	0.0053	0.0053
Wheat_2	NRMSE_SE	Invchi	0.0039	0.0039	0.0039	0.0039
Wheat_2	NRMSE_SE	Logit	0.0043	0.0043	0.0043	0.0043
Wheat_2	NRMSE_SE	Meanp	0.0043	0.0043	0.0043	0.0043
Wheat_2	NRMSE_SE	QM	0.0732	0.0732	0.0732	0.0732
Wheat_2	NRMSE_SE	QM_Invchi	0.0090	0.0090	0.0090	0.0090
Wheat_2	NRMSE_SE	QM_Logit	0.0699	0.0699	0.0699	0.0699
Wheat_2	NRMSE_SE	QM_Meanp	0.0851	0.0851	0.0851	0.0851
Wheat_2	NRMSE_SE	QM_Sumz	0.0659	0.0659	0.0659	0.0659
Wheat_2	NRMSE_SE	SumZ	0.0039	0.0039	0.0039	0.0039
Wheat_3	COR	GBLUP	0.3719	0.3719	0.3719	0.3719
Wheat_3	COR	Invchi	0.3117	0.3117	0.3117	0.3117
Wheat_3	COR	Logit	0.3085	0.3085	0.3085	0.3085
Wheat_3	COR	Meanp	0.3224	0.3224	0.3224	0.3224
Wheat_3	COR	QM	0.3486	0.3486	0.3486	0.3486
Wheat_3	COR	QM_Invchi	0.2866	0.2866	0.2866	0.2866
Wheat_3	COR	QM_Logit	0.2862	0.2862	0.2862	0.2862
Wheat_3	COR	QM_Meanp	0.2808	0.2808	0.2808	0.2808
Wheat_3	COR	QM_Sumz	0.2888	0.2888	0.2888	0.2888
Wheat_3	COR	SumZ	0.3136	0.3136	0.3136	0.3136
Wheat_3	COR_SE	GBLUP	0.0132	0.0132	0.0132	0.0132
Wheat_3	COR_SE	Invchi	0.0107	0.0107	0.0107	0.0107
Wheat_3	COR_SE	Logit	0.0073	0.0073	0.0073	0.0073
Wheat_3	COR_SE	Meanp	0.0106	0.0106	0.0106	0.0106
Wheat_3	COR_SE	QM	0.0194	0.0194	0.0194	0.0194
Wheat_3	COR_SE	QM_Invchi	0.0200	0.0200	0.0200	0.0200
Wheat_3	COR_SE	QM_Logit	0.0204	0.0204	0.0204	0.0204
Wheat_3	COR_SE	QM_Meanp	0.0253	0.0253	0.0253	0.0253
Wheat_3	COR_SE	QM_Sumz	0.0180	0.0180	0.0180	0.0180
Wheat_3	COR_SE	SumZ	0.0097	0.0097	0.0097	0.0097
Wheat_3	NRMSE	GBLUP	0.9299	0.9299	0.9299	0.9299
Wheat_3	NRMSE	Invchi	0.9514	0.9514	0.9514	0.9514
Wheat_3	NRMSE	Logit	0.9533	0.9533	0.9533	0.9533
Wheat_3	NRMSE	Meanp	0.9483	0.9483	0.9483	0.9483
Wheat_3	NRMSE	QM	1.1177	1.1177	1.1177	1.1177
Wheat_3	NRMSE	QM_Invchi	1.1223	1.1223	1.1223	1.1223
Wheat_3	NRMSE	QM_Logit	1.1258	1.1258	1.1258	1.1258
Wheat_3	NRMSE	QM_Meanp	1.1778	1.1778	1.1778	1.1778
Wheat_3	NRMSE	QM_Sumz	1.1217	1.1217	1.1217	1.1217
Wheat_3	NRMSE	SumZ	0.9517	0.9517	0.9517	0.9517
Wheat_3	NRMSE_SE	GBLUP	0.0055	0.0055	0.0055	0.0055
Wheat_3	NRMSE_SE	Invchi	0.0031	0.0031	0.0031	0.0031
Wheat_3	NRMSE_SE	Logit	0.0022	0.0022	0.0022	0.0022
Wheat_3	NRMSE_SE	Meanp	0.0033	0.0033	0.0033	0.0033
Wheat_3	NRMSE_SE	QM	0.0625	0.0625	0.0625	0.0625
Wheat_3	NRMSE_SE	QM_Invchi	0.0635	0.0635	0.0635	0.0635
Wheat_3	NRMSE_SE	QM_Logit	0.0647	0.0647	0.0647	0.0647
Wheat_3	NRMSE_SE	QM_Meanp	0.0823	0.0823	0.0823	0.0823
Wheat_3	NRMSE_SE	QM_Sumz	0.0632	0.0632	0.0632	0.0632
Wheat_3	NRMSE_SE	SumZ	0.0034	0.0034	0.0034	0.0034
Wheat_4	COR	GBLUP	0.3629	0.3629	0.3629	0.3629
Wheat_4	COR	Invchi	0.3311	0.3311	0.3311	0.3311
Wheat_4	COR	Logit	0.3329	0.3329	0.3329	0.3329
Wheat_4	COR	Meanp	0.3409	0.3409	0.3409	0.3409
Wheat_4	COR	QM	0.3505	0.3505	0.3505	0.3505
Wheat_4	COR	QM_Invchi	0.3159	0.3159	0.3159	0.3159
Wheat_4	COR	QM_Logit	0.3326	0.3326	0.3326	0.3326
Wheat_4	COR	QM_Meanp	0.3408	0.3408	0.3408	0.3408
Wheat_4	COR	QM_Sumz	0.3422	0.3422	0.3422	0.3422
Wheat_4	COR	SumZ	0.3414	0.3414	0.3414	0.3414
Wheat_4	COR_SE	GBLUP	0.0149	0.0149	0.0149	0.0149
Wheat_4	COR_SE	Invchi	0.0150	0.0150	0.0150	0.0150
Wheat_4	COR_SE	Logit	0.0165	0.0165	0.0165	0.0165
Wheat_4	COR_SE	Meanp	0.0160	0.0160	0.0160	0.0160
Wheat_4	COR_SE	QM	0.0238	0.0238	0.0238	0.0238
Wheat_4	COR_SE	QM_Invchi	0.0220	0.0220	0.0220	0.0220
Wheat_4	COR_SE	QM_Logit	0.0167	0.0167	0.0167	0.0167
Wheat_4	COR_SE	QM_Meanp	0.0159	0.0159	0.0159	0.0159
Wheat_4	COR_SE	QM_Sumz	0.0157	0.0157	0.0157	0.0157
Wheat_4	COR_SE	SumZ	0.0159	0.0159	0.0159	0.0159
Wheat_4	NRMSE	GBLUP	0.9334	0.9334	0.9334	0.9334
Wheat_4	NRMSE	Invchi	0.9448	0.9448	0.9448	0.9448
Wheat_4	NRMSE	Logit	0.9444	0.9444	0.9444	0.9444
Wheat_4	NRMSE	Meanp	0.9421	0.9421	0.9421	0.9421
Wheat_4	NRMSE	QM	1.1115	1.1115	1.1115	1.1115
Wheat_4	NRMSE	QM_Invchi	1.0890	1.0890	1.0890	1.0890
Wheat_4	NRMSE	QM_Logit	1.0185	1.0185	1.0185	1.0185
Wheat_4	NRMSE	QM_Meanp	1.0222	1.0222	1.0222	1.0222
Wheat_4	NRMSE	QM_Sumz	1.0158	1.0158	1.0158	1.0158
Wheat_4	NRMSE	SumZ	0.9416	0.9416	0.9416	0.9416
Wheat_4	NRMSE_SE	GBLUP	0.0059	0.0059	0.0059	0.0059
Wheat_4	NRMSE_SE	Invchi	0.0047	0.0047	0.0047	0.0047
Wheat_4	NRMSE_SE	Logit	0.0051	0.0051	0.0051	0.0051
Wheat_4	NRMSE_SE	Meanp	0.0050	0.0050	0.0050	0.0050
Wheat_4	NRMSE_SE	QM	0.0758	0.0758	0.0758	0.0758
Wheat_4	NRMSE_SE	QM_Invchi	0.0741	0.0741	0.0741	0.0741
Wheat_4	NRMSE_SE	QM_Logit	0.0138	0.0138	0.0138	0.0138
Wheat_4	NRMSE_SE	QM_Meanp	0.0148	0.0148	0.0148	0.0148
Wheat_4	NRMSE_SE	QM_Sumz	0.0137	0.0137	0.0137	0.0137
Wheat_4	NRMSE_SE	SumZ	0.0050	0.0050	0.0050	0.0050
Wheat_5	COR	GBLUP	0.4367	0.4367	0.4367	0.4367
Wheat_5	COR	Invchi	0.4140	0.4140	0.4140	0.4140
Wheat_5	COR	Logit	0.4157	0.4157	0.4157	0.4157
Wheat_5	COR	Meanp	0.4267	0.4267	0.4267	0.4267
Wheat_5	COR	QM	0.4277	0.4277	0.4277	0.4277
Wheat_5	COR	QM_Invchi	0.3976	0.3976	0.3976	0.3976
Wheat_5	COR	QM_Logit	0.3998	0.3998	0.3998	0.3998
Wheat_5	COR	QM_Meanp	0.4256	0.4256	0.4256	0.4256
Wheat_5	COR	QM_Sumz	0.4046	0.4046	0.4046	0.4046
Wheat_5	COR	SumZ	0.4189	0.4189	0.4189	0.4189
Wheat_5	COR_SE	GBLUP	0.0179	0.0179	0.0179	0.0179
Wheat_5	COR_SE	Invchi	0.0198	0.0198	0.0198	0.0198
Wheat_5	COR_SE	Logit	0.0198	0.0198	0.0198	0.0198
Wheat_5	COR_SE	Meanp	0.0195	0.0195	0.0195	0.0195
Wheat_5	COR_SE	QM	0.0160	0.0160	0.0160	0.0160
Wheat_5	COR_SE	QM_Invchi	0.0209	0.0209	0.0209	0.0209
Wheat_5	COR_SE	QM_Logit	0.0214	0.0214	0.0214	0.0214
Wheat_5	COR_SE	QM_Meanp	0.0188	0.0188	0.0188	0.0188
Wheat_5	COR_SE	QM_Sumz	0.0240	0.0240	0.0240	0.0240
Wheat_5	COR_SE	SumZ	0.0182	0.0182	0.0182	0.0182
Wheat_5	NRMSE	GBLUP	0.9011	0.9011	0.9011	0.9011
Wheat_5	NRMSE	Invchi	0.9128	0.9128	0.9128	0.9128
Wheat_5	NRMSE	Logit	0.9127	0.9127	0.9127	0.9127
Wheat_5	NRMSE	Meanp	0.9067	0.9067	0.9067	0.9067
Wheat_5	NRMSE	QM	1.0787	1.0787	1.0787	1.0787
Wheat_5	NRMSE	QM_Invchi	1.0522	1.0522	1.0522	1.0522
Wheat_5	NRMSE	QM_Logit	1.0517	1.0517	1.0517	1.0517
Wheat_5	NRMSE	QM_Meanp	0.9909	0.9909	0.9909	0.9909
Wheat_5	NRMSE	QM_Sumz	1.0476	1.0476	1.0476	1.0476
Wheat_5	NRMSE	SumZ	0.9098	0.9098	0.9098	0.9098
Wheat_5	NRMSE_SE	GBLUP	0.0097	0.0097	0.0097	0.0097
Wheat_5	NRMSE_SE	Invchi	0.0088	0.0088	0.0088	0.0088
Wheat_5	NRMSE_SE	Logit	0.0093	0.0093	0.0093	0.0093
Wheat_5	NRMSE_SE	Meanp	0.0090	0.0090	0.0090	0.0090
Wheat_5	NRMSE_SE	QM	0.0527	0.0527	0.0527	0.0527
Wheat_5	NRMSE_SE	QM_Invchi	0.0605	0.0605	0.0605	0.0605
Wheat_5	NRMSE_SE	QM_Logit	0.0612	0.0612	0.0612	0.0612
Wheat_5	NRMSE_SE	QM_Meanp	0.0211	0.0211	0.0211	0.0211
Wheat_5	NRMSE_SE	QM_Sumz	0.0715	0.0715	0.0715	0.0715
Wheat_5	NRMSE_SE	SumZ	0.0080	0.0080	0.0080	0.0080
Wheat_6	COR	GBLUP	0.5307	0.5307	0.5307	0.5307
Wheat_6	COR	Invchi	0.5167	0.5167	0.5167	0.5167
Wheat_6	COR	Logit	0.5218	0.5218	0.5218	0.5218
Wheat_6	COR	Meanp	0.5206	0.5206	0.5206	0.5206
Wheat_6	COR	QM	0.5109	0.5109	0.5109	0.5109
Wheat_6	COR	QM_Invchi	0.5001	0.5001	0.5001	0.5001
Wheat_6	COR	QM_Logit	0.5192	0.5192	0.5192	0.5192
Wheat_6	COR	QM_Meanp	0.5200	0.5200	0.5200	0.5200
Wheat_6	COR	QM_Sumz	0.4970	0.4970	0.4970	0.4970
Wheat_6	COR	SumZ	0.5183	0.5183	0.5183	0.5183
Wheat_6	COR_SE	GBLUP	0.0092	0.0092	0.0092	0.0092
Wheat_6	COR_SE	Invchi	0.0107	0.0107	0.0107	0.0107
Wheat_6	COR_SE	Logit	0.0095	0.0095	0.0095	0.0095
Wheat_6	COR_SE	Meanp	0.0093	0.0093	0.0093	0.0093
Wheat_6	COR_SE	QM	0.0164	0.0164	0.0164	0.0164
Wheat_6	COR_SE	QM_Invchi	0.0231	0.0231	0.0231	0.0231
Wheat_6	COR_SE	QM_Logit	0.0096	0.0096	0.0096	0.0096
Wheat_6	COR_SE	QM_Meanp	0.0088	0.0088	0.0088	0.0088
Wheat_6	COR_SE	QM_Sumz	0.0191	0.0191	0.0191	0.0191
Wheat_6	COR_SE	SumZ	0.0112	0.0112	0.0112	0.0112
Wheat_6	NRMSE	GBLUP	0.8498	0.8498	0.8498	0.8498
Wheat_6	NRMSE	Invchi	0.8631	0.8631	0.8631	0.8631
Wheat_6	NRMSE	Logit	0.8598	0.8598	0.8598	0.8598
Wheat_6	NRMSE	Meanp	0.8582	0.8582	0.8582	0.8582
Wheat_6	NRMSE	QM	0.9863	0.9863	0.9863	0.9863
Wheat_6	NRMSE	QM_Invchi	0.9590	0.9590	0.9590	0.9590
Wheat_6	NRMSE	QM_Logit	0.8994	0.8994	0.8994	0.8994
Wheat_6	NRMSE	QM_Meanp	0.9017	0.9017	0.9017	0.9017
Wheat_6	NRMSE	QM_Sumz	0.9536	0.9536	0.9536	0.9536
Wheat_6	NRMSE	SumZ	0.8606	0.8606	0.8606	0.8606
Wheat_6	NRMSE_SE	GBLUP	0.0059	0.0059	0.0059	0.0059
Wheat_6	NRMSE_SE	Invchi	0.0062	0.0062	0.0062	0.0062
Wheat_6	NRMSE_SE	Logit	0.0058	0.0058	0.0058	0.0058
Wheat_6	NRMSE_SE	Meanp	0.0052	0.0052	0.0052	0.0052
Wheat_6	NRMSE_SE	QM	0.0676	0.0676	0.0676	0.0676
Wheat_6	NRMSE_SE	QM_Invchi	0.0648	0.0648	0.0648	0.0648
Wheat_6	NRMSE_SE	QM_Logit	0.0107	0.0107	0.0107	0.0107
Wheat_6	NRMSE_SE	QM_Meanp	0.0089	0.0089	0.0089	0.0089
Wheat_6	NRMSE_SE	QM_Sumz	0.0509	0.0509	0.0509	0.0509
Wheat_6	NRMSE_SE	SumZ	0.0064	0.0064	0.0064	0.0064

## References

[1] Meuwissen T.H., Hayes B.J., Goddard M.E. (2001). Prediction of total genetic value using genome-wide dense marker maps. Genetics.

[2] Crossa J., Pérez-Rodríguez P., Cuevas J., Montesinos-López O., Jarquín D., de Los Campos G., Burgueño J., González-Camacho J.M., Pérez-Elizalde S., Beyene Y. (2017). Genomic selection in plant breeding: Methods, models, and perspectives. Trends Plant Sci..

[3] Heffner E.L., Sorrells M.E., Jannink J.L. (2009). Genomic selection for crop improvement. Crop Sci..

[4] Varshney R.K., Roorkiwal M., Sorrells M.E. (2017). Genomic selection for crop improvement: An introduction. Genomic Selection for Crop Improvement: New Molecular Breeding Strategies for Crop Improvement.

[5] Xu Y., Liu X., Fu J., Wang H., Wang J., Huang C., Prasanna B.M., Olsen M.S., Wang G., Zhang A. (2020). Enhancing genetic gain through genomic selection: From livestock to plants. Plant Commun..

[6] Voss-Fels K.P., Cooper M., Hayes B.J. (2019). Accelerating crop genetic gains with genomic selection. Theor. Appl. Genet..

[7] Rutkoski J., Poland J., Mondal S., Autrique E., Pérez L.G., Crossa J., Reynolds M., Singh R. (2016). Canopy temperature and vegetation indices from high-throughput phenotyping improve accuracy of pedigree and genomic selection for grain yield in wheat. G3 Genes Genomes Genet..

[8] VanRaden P.M. (2008). Efficient methods to compute genomic predictions. J. Dairy Sci..

[9] Fernando R.L., Gianola D. (1986). Optimal properties of the conditional mean as a selection criterion. TAG Theor. Appl. Genet. Theor. Angew. Genet..

[10] Robinson G.K. (1991). That BLUP is a Good Thing: The Estimation of Random Effects. Stat. Sci..

[11] Cannon A.J., Sobie S.R., Murdock T.Q. (2015). Bias Correction of GCM Precipitation by Quantile Mapping: How Well Do Methods Preserve Changes in Quantiles and Extremes?. J. Clim..

[12] Tarr G., Müller S., Weber N.C. (2016). Robustness and outlier detection in genomic prediction. BMC Bioinform..

[13] Gudmundsson L., Bremnes J.B., Haugen J.E., Engen-Skaugen T. (2012). Technical Note: Downscaling RCM precipitation to the station scale using statistical transformations—A comparison of methods. Hydrol. Earth Syst. Sci..

[14] Li H., Sheffield J., Wood E.F. (2010). Bias correction of monthly precipitation and temperature fields from Intergovernmental Panel on Climate Change AR4 models using equidistant quantile matching. J. Geophys. Res. Atmos..

[15] Feng S., Zhang H., Tong X., Wang Y., Liu B. (2021). Application of quantile mapping bias correction in remote sensing hydrology. Remote Sens..

[16] Hodge V.J., Austin J. (2004). A survey of outlier detection methodologies. Artif. Intell. Rev..

[17] Zhou L., Ding X., Zhang Q., Wang Y., Lund M.S., Su G. (2014). Influence of outliers on accuracy of genomic prediction for feed efficiency traits in dairy cattle. J. Dairy Sci..

[18] González-Recio O., Rosa G.J.M., Gianola D. (2014). Machine learning methods and predictive ability metrics for genome-wide prediction of complex traits. J. Dairy Sci..

[19] Heslot N., Akdemir D., Sorrells M.E., Jannink J.L. (2014). Integrating environmental covariates and crop modeling into the genomic selection framework to predict genotype by environment interactions. Theor. Appl. Genet..

[20] Spindel J.E., McCouch S.R. (2016). When more is better: How data sharing would accelerate genomic selection of crop plants. New Phytol..

[21] Montesinos-López O.A., Pierre C.S., Gezan S.A., Bentley A.R., Mosqueda-González B.A., Montesinos-López A., van Eeuwijk F., Beyene Y., Gowda M., Gardner K. (2023). Optimizing sparse testing for genomic prediction of plant breeding crops. Genes.

[22] Montesinos-López O.A., Mosqueda-González B.A., Salinas-Ruiz J., Montesinos-López A., Crossa J. (2023). Sparse multi-trait genomic prediction under balanced incomplete block design. Plant Genome.

[23] Montesinos-López O.A., Herr A.W., Crossa J., Montesinos-López A., Carter A.H. (2024). Enhancing winter wheat prediction with genomics, phenomics and environmental data. BMC Genom..

[24] Hickey J.M., Chiurugwi T., Mackay I., Powell W. (2017). Genomic prediction unifies animal and plant breeding programs to form platforms for biological discovery. Nat. Genet..

[25] Wang X., Xu Y., Hu Z., Xu C. (2018). Genomic selection methods for crop improvement: Current status and prospects. Crop J..

[26] R Core Team (2024). R: A Language and Environment for Statistical Computing. R Foundation for Statistical Computing.

[27] Pérez P., de Los Campos G. (2014). Genome-wide regression and prediction with the BGLR statistical package. Genetics.

[28] Tong Y., Gao X., Han Z., Xu Y., Xu Y., Giorgi F. (2021). Bias correction of temperature and precipitation over China for RCM simulations using the QM and QDM methods. Clim. Dyn..

[29] Piani C., Haerter J.O., Coppola E. (2010). Statistical bias correction for daily precipitation in regional climate models over Europe. Theor. Appl. Climatol..

[30] Budhlakoti N., Rai A., Mishra D.C. (2020). Statistical approach for improving genomic prediction accuracy through efficient diagnostic measure of influential observation. Sci. Rep..

[31] Won S., Morris N., Lu Q., Elston R.C. (2009). Choosing an optimal method to combine P-values. Stat. Med..

[32] Mudholkar G.S., George E.O., Rustagi J. (1979). The logit method for combining probabilities. Symposium on Optimizing Methods in Statistics.

[33] Sutton A.J., Abrams K.R., Jones D.R., Sheldon T.A., Song F. (2000). Methods for Meta-Analysis in Medical Research.

[34] Stouffer S.A., Suchman E.A., DeVinney L.C., Star S.A., Williams R.M. (1949). The American Soldier: Adjustment During Army Life. (Studies in Social Psychology in World War ii), Vol. 1.

[35] Montesinos López O.A., Montesinos-López A., Crossa J. (2022). Multivariate Statistical Machine Learning Methods for Genomic Prediction.

